# Self-Induced Acidification of Fuel Ethanol and Its Role in Corrosion: Mitigation via Ion-Exchange Resins

**DOI:** 10.1021/acsomega.6c03466

**Published:** 2026-06-26

**Authors:** Chrysttian M. S. Garcia, Estevan D Cruz, Francisco D. Vezaro, Alex Schulz, Larissa Rossignoli Mariano, Fernando Luiz Windlin, Ederson R. Abaide, Flávio D. Mayer

**Affiliations:** † Laboratory of Biomass and Biofuels, 28118 Federal University of Santa Maria, Roraima Avenue, 1000, Santa Maria, RS 97105-900, Brazil; ‡ Department of Chemical Engineering, Federal University of Santa Maria, Roraima Avenue, 1000, Santa Maria, RS 97105-900, Brazil; § Marelli Sistemas Automotivos, Emancipação Avenue, 801, Hortolândia, SP 13184-654, Brazil

## Abstract

The presence of organic acids in fuel ethanol can significantly increase the corrosivity of automotive fuel systems. This study evaluated the relationship between ethanol composition and corrosion of stainless steel fuel injector valves and investigated the removal of acetic acid from fuel ethanol using ion-exchange resins. Long-term immersion assays (90 days at 70 °C) were performed and revealed no visible corrosion in anhydrous ethanol, while hydrated ethanol promoted severe corrosion accompanied by acetic acid concentrations exceeding 900 mg·L^–1^. Commercial ethanol samples also showed corrosion when total acidity increased up to 498.6 mg·L^–1^. Synthetic ethanol tests confirmed that increasing acetic acid concentrations intensified corrosion and that ethanol–metal interactions can promote acid formation, reaching up to 372.6 mg·L^–1^ even in initially acid-free solutions. To mitigate this problem, the adsorption performance of Amberlite IRA-67 and IRA-96 resins was evaluated. IRA-67 exhibited superior performance, reaching adsorption equilibrium within 60–90 min and maximum adsorption capacities between 14 and 25 mg·g^–1^. The resin maintained good selectivity in the presence of competing acids and retained about 85% of its adsorption capacity after five regeneration cycles. In real fuel ethanol samples, acetic acid removal ranged from 30 to 41%, reducing concentrations below regulatory limits. Continuous fixed-bed experiments showed initial removal efficiencies above 95%, with breakthrough occurring after approximately 50 min. The results confirmed the feasibility of ion-exchange technology for fuel ethanol purification and can be used to design a filter to be used in gas stations or in an automotive fuel injection system.

## Graphical abstract

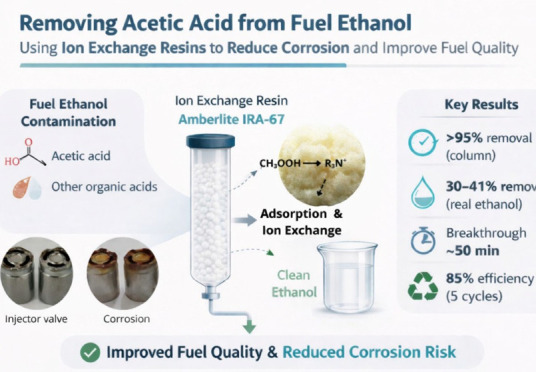

## 1 Introduction

Faced with the challenges of reducing CO_2_ emissions into the atmosphere and concerns about the decline of fossil resources, the increasing use of renewable fuel sources, such as ethanol, is notable because it reduces greenhouse gas emissions by 40–62% compared to fossil fuels.^1^ The United States of America and Brazil are the world’s largest ethanol producers,^2^ accounting for 61.40 billion and 37 billion liters in 2024, respectively.^3,4^

Impurities, water, and organic acids can be found in ethanol obtained from the biomass. Some compounds present in ethanol can cause corrosion of engine components, such as water, methanol, acid residues, chlorides, and sulfates.^5^ Because of its high hydrophilicity, ethanol can increase the water content in automotive fuel systems, and the acetic acid produced from the oxidation of organic compounds can promote increased corrosion of metal components.^6,7^ Inconveniently, some acetic acid can remain in ethanol after the distillation and dehydration processes or can be formed after relatively long periods of storage.^8^ Other organic acids commonly found in ethanol fuel are formic, propionic, and *n*-butyric acid.^9^

In parallel with the increased use of ethanol as a renewable fuel, many studies have evaluated the corrosive behavior of this biofuel on various metals and alloys. Most of the research evaluates the corrosion of carbon steel or low-alloy steels in contact with ethanol or ethanol–gasoline blends.^6,10,11^ Corrosion of carbon steel from fuel ethanol with different water, chloride, and acetic acid contents was assessed in a previous study,^12^ demonstrating the strong relevance of water content in corrosion induction. Furthermore, it is noteworthy that higher acetic acid contents in ethanol led to a larger attack area during the corrosion process.^10^

To remove compounds that impart corrosive characteristics to fuel ethanol, different methods have been used, such as supercritical fluid extraction, ion exchange, electrodialysis, and methods using membranes in pressurized systems.^13,14^ Some methods, such as neutralization and distillation, are widely used industrially, but they face economic and environmental problems, since the low concentrations of acetic acid in fermented broths require significant energy consumption. The presence of phosphates, chlorides, sulfates, and proteins, which can interfere with the purification stage, must also be considered in the downstream separation process.^15^

One technology that requires low energy consumption for the ethanol purification stage is ion exchange (IEX). This separation technology uses IEX resins, composed of a polymer with positively or negatively charged functional groups, to remove diluted contaminants. The removal of acids from aqueous and organic media using ion-exchange resins has been considered an efficient and economical process, including applications in bioseparations of organic acids, such as acetic acid, present in aqueous fermentation broths.^16,17^ However, the literature presents a gap regarding the use of ion-exchange resins for acetic acid removal from fuel ethanol, with studies in this area still being limited.

Given the present context and the need to remove contaminants in fuel ethanol in order to avoid corrosion problems in automotive engine parts, this study aims to evaluate the corrosive effect of corn-derived fuel ethanol samples obtained from different regions of Brazil on stainless steel fuel injector valves, as well as the composition of these samples, relating the effect of water and organic acid (acetic and formic) contents on the corrosion process. On the basis of this assessment and information from recent literature studies, the adsorption capacity of IRA-67 and IRA-96 resins for the removal of acetic acid from fuel ethanol was assessed.

## 2 Materials and Methods

The experimental procedures adopted in this study are described in detail in the . A brief overview of the main methodological steps is provided in the following subsections.

### 2.1 Corrosion Assay

Corrosion assays were conducted by immersing stainless steel fuel injector valves in ethanol samples for 90 days at 70 ± 3 °C, following the guidelines of ASTM G31-21.^19^ Real fuel ethanol samples were collected from distilleries and fuel stations in the states of Mato Grosso, Goiás, and Mato Grosso do Sul, in both anhydrous and hydrated forms. Additionally, synthetic hydrated ethanol solutions (95% v/v) were prepared in the laboratory with defined initial acetic acid concentrations of 46.8, 91.3, and 169.8 mg·L^–1^, along with a control solution without acetic acid addition.

### 2.2 Adsorption

Adsorption experiments were performed in batch mode using Amberlite IRA-67 and IRA-96 resins, evaluating adsorbent dosage, kinetics, equilibrium isotherms, thermodynamic parameters, competitive adsorption, and resin regeneration. Continuous flow adsorption tests were conducted in a stainless steel fixed-bed column (1.0 cm internal diameter, 12.5 cm height) at 4 MPa and 6.5 mL·min^–1^.

### 2.3 Analytical Methods

Organic acid concentrations in all samples were quantified by gas chromatography (GC) equipped with a flame ionization detector (FID) based on standard NBR 16041. Resin characterization was performed by Fourier transform infrared spectroscopy (FT-IR).

## 3 Results and Discussion

### 3.1 Corrosion Assay and Ethanol Composition

#### 3.1.1 Real Sample

For the corrosion assay performed on the stainless steel fuel injector valves, evaluating the effect of anhydrous and hydrated corn ethanol obtained from a refinery in the central-west of Brazil. Figure 1 shows the images of the fuel injector valve corrosion assay after 90 days and the changes in the composition of ethanol. In a corrosion assay performed on stainless steel fuel injector valves submerged in samples of anhydrous corn ethanol, visual inspection revealed no signs of corrosion on these parts (Figure 1a). The surface of the stainless steel fuel injector valves remained visually intact, with a homogeneous appearance and preserved metallic shine, without evidence of deposits, stains, or color alteration, which suggests good stability of the medium and low propensity for oxidation or degradation processes. When evaluating the anhydrous corn ethanol in which these materials were immersed, it can be observed that the organic acid levels did not present statistically significant variations from the beginning of the assay to the end (90 days) (Figure 1b), with a total acidity of 117.2 mg·L^–1^ initially, reaching a value of 186.2 mg·L^–1^ in 90 days. This value is higher than the specified limit for acidity in ethanol (30 mg·L^–1^) according to the ANP resolution no. 907, of Nov 18, 2022.^31^

**Figure 1:**
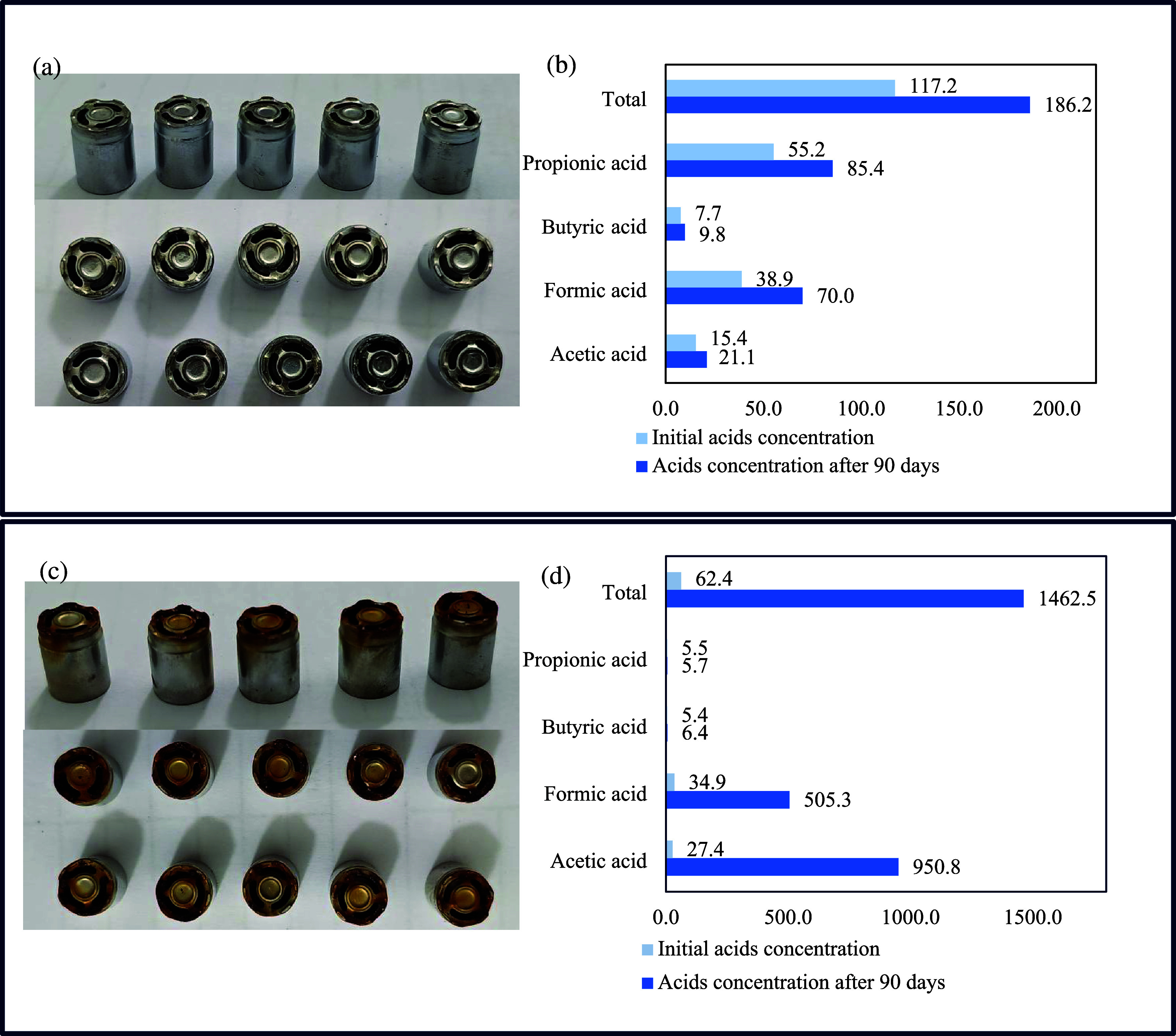
Stainless steel fuel injector valves tested with anhydrous ethanol (a, b) and hydrated ethanol (c, d) samples collected from different distilleries in different regions of Brazil, showing acid concentrations measured before and after corrosion assay.

On the other hand, samples in hydrated corn ethanol presented clear signs of aggressive corrosion in the metallic parts, as can be seen in Figure 1c. The surfaces exhibited evident yellowish coloration in the upper part, in addition to the presence of a solid precipitate adhering to the upper region. This visual behavior reinforces the presence of intense corrosive reactions. Furthermore, a significant increase in the concentration of organic acids in the solution was noted (Figure 1d), with emphasis on acetic acid, which exceeded 900 mg·L^–1^, and formic acid, which also presented considerable growth. These two acids were mainly responsible for the increase in solution acidity. In contrast, propionic and butyric acid remained practically constant between different samples, indicating lower reactivity under the analyzed conditions. Acetic acid plays a critical role in carbon steel corrosion in fuel ethanol, being one of the main agents that promote pitting corrosion through iron acetate formation and alteration of the surface oxide film samples.^18^ This can be seen in the results obtained from real ethanol.

Another study reported in the literature evaluated the effect of acetic acid content in ethanol on corrosion in steel parts through immersion tests conducted for 8 days at room temperature, where it was found that the increase in acidity increases both pitting and general corrosion.^12^ In a similar investigation, researchers studied the combined effects of acetic acid, formic acid, and water in ethanol on steel parts subjected to corrosion tests for 30 days.^18^ According to the study, materials submerged in ethanol samples containing 1120 mg·g^–1^ of acetic acid exhibited more severe pitting corrosion. The research found that no distinguishable corrosion spots were observed on the carbon steel surface when exposed to ethanol with less than 1% by volume of water. However, the investigation demonstrated that as the water content increased from 5 to 10% by volume, visual corrosion became apparent, with the corrosion area increasing drastically by up to 5 times.

In general, the results of this study, shown in Figure 1, evidence the difference in behavior between the two types of ethanol evaluated. While anhydrous ethanol proved to be a more stable and less aggressive medium to metallic surfaces, hydrated ethanol indicated a tendency toward chemical degradation and formation of corrosive compounds, compromising the integrity of metallic parts exposed to this type of fuel.^12^

The higher formation of organic acids observed in hydrated ethanol can be explained by an oxidative path that is well-established in the literature. Ethanol undergoes oxidation by dissolved atmospheric oxygen, forming acetaldehyde as an intermediate, which in turn undergoes autoxidation, resulting in the formation of acetic acid.^32,33^ Water acts as a facilitator of this process, as its presence increases the ionic conductivity of the solution, promotes electrochemical reactions at the metal–solution interface, and intensifies the instability of the passive film, making the medium more susceptible to corrosion.^12^ In anhydrous ethanol, the absence of water significantly limits these degradation pathways, which is consistent with the chemical stability and absence of visual corrosion observed in the anhydrous samples in this study.^12,18^

After the primary assay, commercial ethanol samples were collected from the refinery and fuel stations in three states in Brazil. Mato Grosso (MT), Goiás (GO), and Rio Grande do Sul (RS) were analyzed with the objective of comparing behavior between regions. The MT and GO samples were identified as corn ethanol, while the origin of the RS ethanol was not specified. The evaluation performed was similar to the previous one, verifying the occurrence of corrosion in the fuel injector valves and the composition of ethanol samples (Figure 2).

**Figure 2:**
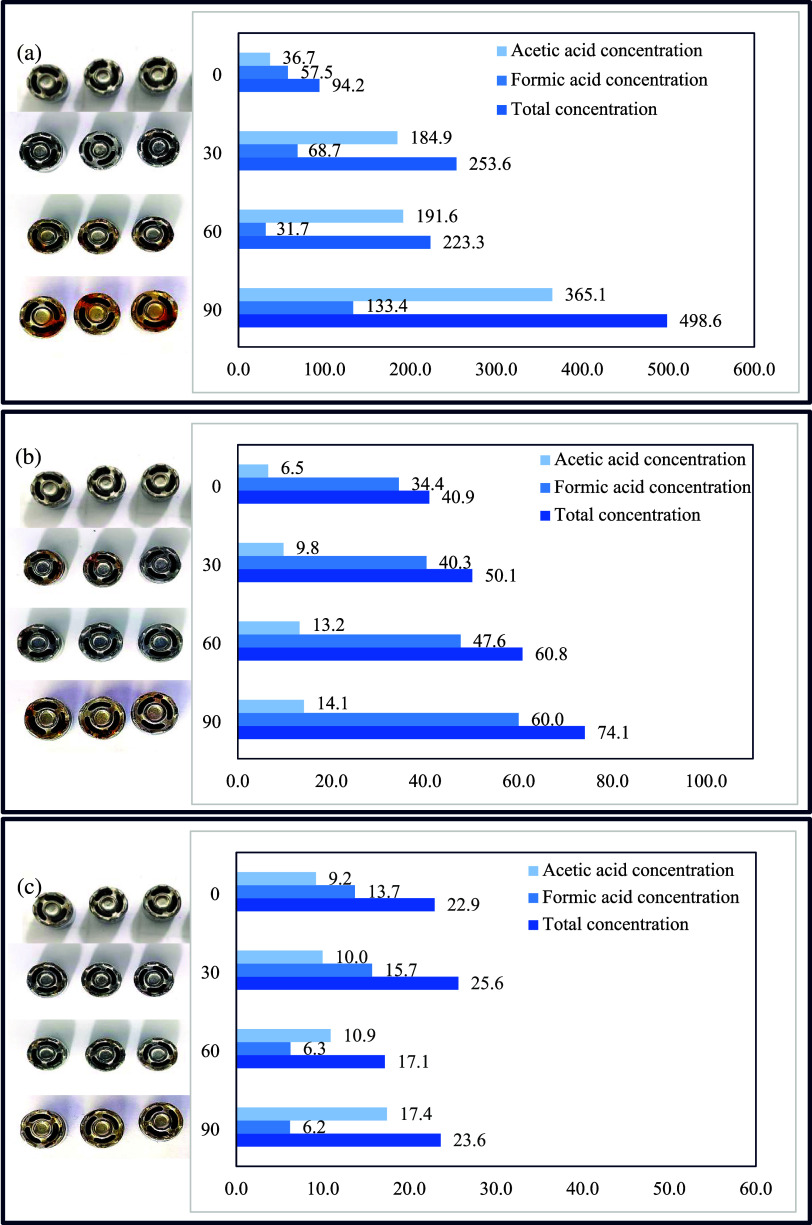
Stainless-steel fuel injector valves used in assays with hydrated ethanol from the states of Mato Grosso (a), Goiás (b), and Rio Grande do Sul (c), evaluated in four exposure periods, with their respective acid concentrations.

The MT and GO samples present corrosion in the metallic parts, as illustrated in Figure 2a,b. In both cases, it was possible to observe darkening and progressive deterioration of metallic surfaces during the assay time periods. Corrosion was more severe in the ethanol sample from Mato Grosso, with evident signs already at 60 days, becoming even more accentuated at 90 days. In the ethanol from the GO state, the visual effects of corrosion were more perceptible at the end of 90 days (Figure 2b).

These samples also presented an expressive increase in the concentration of organic acids throughout the period evaluated, as demonstrated in Figure 2a–c. In the case of the ethanol sample from MT, total acidity increased from 94.2 mg·L^–1^ (day 0) to 498.6 mg·L^–1^ (day 90), with emphasis on the significant elevation of acetic acid (36.7–365.1 mg·L^–1^). In GO ethanol, total acidity went from 40.9 mg·L^–1^ (day 0) to 74.1 mg·L^–1^ (90 days), also with relevant growth in monitored acids.

On the other hand, the ethanol sample from Rio Grande do Sul, of undefined origin, did not present visual signs of corrosion in the metallic parts (Figure 2c). In the ethanol sample from this state, there was a slight change in total acid composition, from 22.9 mg·L^–1^ (0 days) to 23.6 mg·L^–1^ (90 days) (Figure 2c), indicating low corrosive activity and minimal formation of organic acids over time. This agrees with what has been reported in the other study,^12^ where fuel ethanol with high concentrations of acetic acid promotes a corrosion process in carbon steel.

#### 3.1.2 Ethanol Synthetic Samples

Based on the results obtained with real samples (Section 3.1.1), which presented an expressive increase in acetic acid concentration, ethanol synthetic solutions prepared in the laboratory containing different initial concentrations of this acid were assessed. The objective was to monitor, under controlled experimental conditions (temperature of 70 ± 3 °C, closed system with condenser to prevent evaporation losses, total immersion time of 90 days, and defined initial acetic acid concentrations), the behavior of metallic corrosion and the evolution of acetic acid formation over time. As illustrated in Figure 3, all stainless steel fuel injector valves exposed to ethanol synthetic solutions presented some degree of corrosion. The intensity varied according to the initial acetic acid concentration, being observed from the formation of deposits adhered to the surface to localized darkening and loss of metallic shine, mainly in the upper regions of the parts.

**Figure 3:**
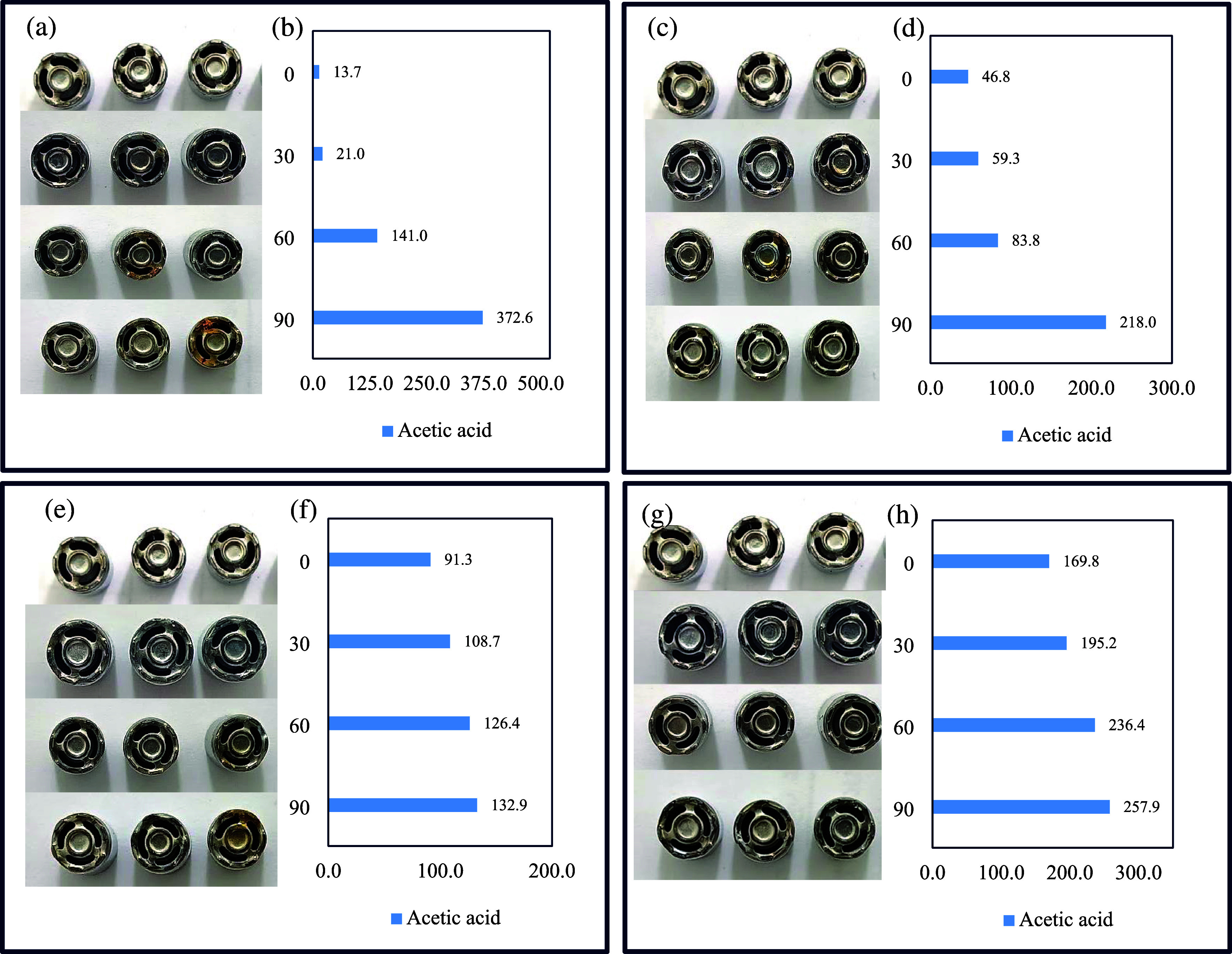
Stainless steel fuel injector valves used in assays with synthetic ethanol solutions: control solution 13.7 mg·L^–1^ (a, b), 46.8 mg·L^–1^ (c, d), 91.3 mg·L^–1^ (e, f), and 169.7 mg·L^–1^ (g, h), along with their respective acetic acid concentrations.

Regardless of initial concentration, all solutions presented a significant increase in acetic acid levels at the end of the exposure period, as shown in Figure 3. The solution with an initial concentration of 168.8 mg·L^–1^ reached 257.9 mg·L^–1^, being the second highest value recorded. Following, the solution with 46.8 mg·L^–1^ reached 218.0 mg·L^–1^, while the 91.3 mg·L^–1^ solution reached 132.9 mg·L^–1^.

Curiously, the control solution, which did not receive acetic acid addition and contained only compounds naturally present in ethanol, was the one that presented the greatest concentration, reaching 372.6 mg·L^–1^ of acetic acid. This result reinforces the hypothesis that the contact between ethanol and metal itself is capable of triggering degradation reactions that lead to the spontaneous formation of acidic compounds.^34^ Such behavior suggests that metal acts as a catalyst in the oxidation of ethanol or its byproducts, making the ethanol–metal system chemically unstable over time, even in the absence of added contaminants.^12,18^

The nonlinear behavior observed in acetic acid accumulation across the different synthetic solutions may be attributed to a combination of factors. In the control solution, the absence of added acetic acid allowed ethanol oxidation to proceed more freely, favoring greater net acid generation via the acetaldehyde intermediate.^32,33^ In solutions with higher initial acetic acid concentrations, the increased acidity of the medium from the outset may have partially inhibited further oxidation reactions, resulting in a lower net acid accumulation. The variability in dissolved oxygen levels across different flasks, which were not systematically controlled in this study, may have further contributed to the observed differences in reaction rates.^35^

From a visual standpoint, it was also possible to identify differences associated with the initial acid concentration. In solutions with higher initial acid content, fuel injector valves presented greater darkening and a greater amount of precipitated material, indicating more severe corrosion. In less-concentrated solutions, effects were less intense, limited to loss of metallic shine and formation of a thin oxidation film.

In general, the results obtained indicate a clear relationship between the level of acetic acid formation and metallic corrosion advancement over time. The generation of acidic compounds in initially neutral solutions, combined with corrosion intensification in environments with greater contaminant loads, reinforces the aggressive character of fuel ethanol when heated and exposed to stainless steel materials. These findings evidence the importance of rigorously monitoring ethanol quality, especially regarding acidity and the presence of corrosive substances. Additionally, they demonstrate that factors such as initial composition and ethanol usage history are determining factors for their chemical stability and corrosive potential in real systems.

In addition to acetic acid, formic acid also showed a significant increase in hydrated ethanol samples. Formic acid can originate from the further oxidation of acetaldehyde along the same oxidative chain responsible for acetic acid formation, as well as from the degradation of other organic components present in the biomass feedstock.^32,36^ Both acetic and formic acids are known to contribute to the corrosion of metallic components in fuel ethanol systems, as they promote acidification of the medium and facilitate the dissolution of the protective oxide film on metal surfaces.^12^ In contrast, propionic and butyric acids remained practically constant throughout the experiments, suggesting that these compounds are primarily residual from the fermentation process and are not significantly generated through ethanol-metal interactions under the conditions studied.

Although this study focused on stainless-steel fuel injector valves, the findings have broader implications for other metallic materials commonly used in fuel systems. Carbon steel and zinc-based alloys, such as zamak, are particularly susceptible to corrosion in fuel ethanol environments, with corrosion rates reported to be up to 400 times higher in solutions containing water and ionic impurities compared to anhydrous ethanol.^35^ Copper has also been identified as highly susceptible to corrosion in ethanol-containing fuels.^10^ Given that acetic acid is a key driver of corrosive processes across these different material systems, the reduction in the acetic acid concentration achieved through ion exchange treatment is expected to contribute to corrosion mitigation in a broader range of automotive fuel system components.

#### 3.1.3 Limitations

It is important to highlight some limitations of the corrosion assays used in this study. The corrosion assessment relied on visual inspection of the fuel-injector valve surfaces combined with chemical analysis of the ethanol samples over time. Quantitative corrosion measurements, such as mass loss, surface roughness, and pit density, were not performed, which constrains a more precise characterization of the corrosion extent. Additionally, corrosion assays were conducted without replication, which limited the statistical evaluation of the results. Furthermore, the water content and dissolved oxygen were not systematically controlled during the experiments. Both variables are known to significantly influence corrosion behavior: water increases the ionic conductivity of the solution and promotes electrochemical reactions at the metal-solution interface, while dissolved oxygen acts as a cathodic reactant capable of accelerating corrosion kinetics.^12^ The potential influence of these variables on the observed results should therefore be taken into account when interpreting the findings of this study. Future work should incorporate quantitative corrosion metrics and systematic control of these parameters to allow for a more comprehensive characterization of the corrosion process.

### 3.2 Adsorption Tests

#### 3.2.1 Adsorbent Dosage

The adsorbent dosage is one of the most relevant parameters in the adsorption process performance, directly influencing both removal efficiency and system economic viability. As presented in Figure 4a, the increase in adsorbent dosage (IRA-67 resin) from 0.5 to 5 g·L^–1^ resulted in a significant rise in removal efficiency that varied from approximately 30% to about 58% for IRA-67 resin. This behavior can be attributed to the greater availability of active adsorption sites, which favor interaction with acetic acid molecules present in the solution.^37^ However, an inverse trend was observed for the adsorption capacity (*q_t_*) of IRA-67 resin. The *q_t_* values decreased from approximately 17 mg·g^–1^ at the lowest dosage to approximately 3 mg·g^–1^ at the highest concentration evaluated. This phenomenon occurs when the number of available sites exceeds the amount of solute in the solution, resulting in adsorbent underutilization.

**Figure 4:**
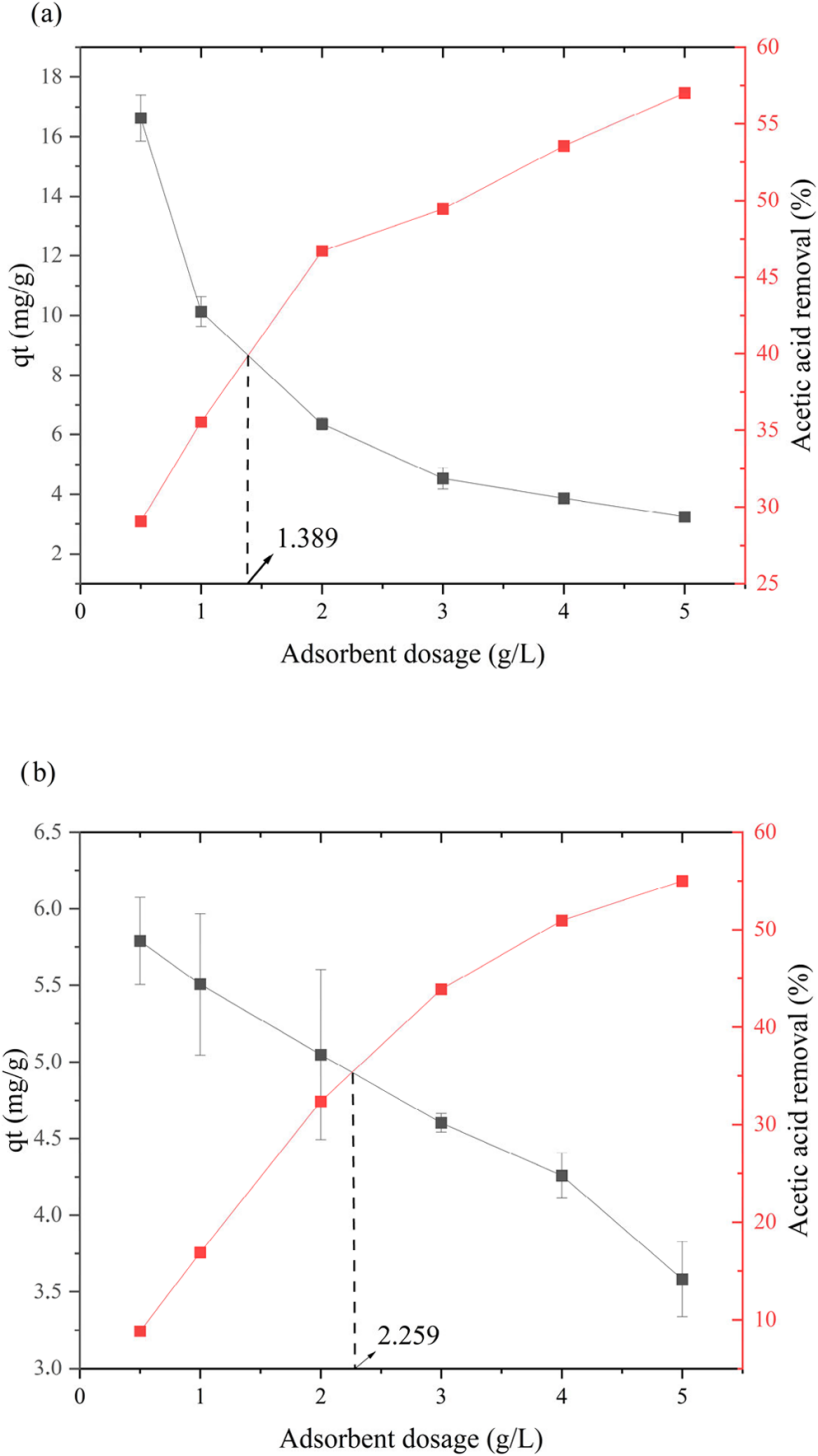
Effect of dosage on acetic acid adsorption onto ion-exchange resin sample, IRA-67 (a), IRA-96 (b) (*C*_0_ = 20 mg·L^–1^, 25 °C, 150 rpm, and 24 h).

The dosage effect of IRA-96 resin is presented in Figure 4b. As the dosage increased from 0.5 to 5 g·L^–1^, the acetic acid removal efficiency progressively increased, reaching about 55% at the highest dosage evaluated. On the other hand, the adsorption capacity (*q_t_*) initially increased from approximately 3.5–5.7 mg·g^–1^. This profile indicates that, despite the increase in overall efficiency, the specific utilization of the adsorbent is reduced at high concentrations due to partial saturation and resin underutilization.^38^

Compared to IRA-67, the IRA-96 resin presented inferior performance in terms of adsorption capacity. However, it achieved equally high removal efficiencies, which demonstrates its potential, especially when the priority is maximizing removal rather than the yield per gram of resin. Among the resins tested to remove acetic acid in an ethanolic medium, the one with tertiary amine functional groups (IRA-67) was prominent, with the best performance in adsorption. This can be explained by the ability of these groups to form specific interactions with the acid, especially through weak and reversible hydrogen bonds, which favor both selective adsorption and resin regeneration. The resin functionalized with a polyamide group (IRA-96) had inferior performance, possibly because its electronic accessibility is lower and its geometry does not favor the formation of effective interactions with acidic hydrogen. Thus, the results indicate that tertiary amine groups create more favorable conditions for selective adsorption of acetic acid, proving to be the most efficient functionality among the evaluated resins.^39^

The superior performance of IRA-67 resin (weak base, tertiary amine) over IRA-96 resin observed in this work is consistent with findings reported in the literature,^20^ who compared weak base resins (330 and D301R) and strong base resins (201 × 7 and D201) for acetic acid removal from ethanolic solutions. The weak base resin 330, containing secondary, tertiary, and quaternary amine functional groups, exhibited the highest dynamic adsorption capacity, significantly outperforming the strong base resins.^21,22^ These results reinforce that weak base resins with amine functional groups are more suitable for carboxylic acid adsorption from organic media.

The superior adsorption capacity of IRA-67 can be further understood in terms of the basicity of its tertiary amine functional groups and their role in the adsorption mechanism. In nonaqueous media such as ethanol, where ionic dissociation is negligible, adsorption occurs predominantly through the formation of hydrogen-bonded complexes between the lone pair of electrons on the nitrogen atom of the tertiary amine and the acidic proton of acetic acid, a Lewis acid–base interaction.^39^ Tertiary amines offer accessible nitrogen lone pairs that readily participate in this interaction, favoring selective and reversible adsorption, while quaternary ammonium groups exhibit greater steric hindrance around the nitrogen atom, limiting effective hydrogen bond formation with the acidic proton and resulting in lower adsorption capacity.^20,39^ Weak-base resins with tertiary amine functional groups, including IRA-67, have been reported to exhibit the highest acetate adsorption capacity among different resin types, outperforming strong-base resins containing quaternary ammonium groups.^17^ These findings suggest a direct correlation among amine type, accessibility of the nitrogen lone pair, and adsorption capacity for acetic acid in ethanolic systems.

Based on experimental data, IRA-67 resin was considered the best and was therefore used in the subsequent assay. The optimal dosage of IRA-67 was established as 1.389 g·L^–1^, the point at which the highest *q_t_* value was observed together with satisfactory removal efficiency (∼40%), before the sharp drop in capacity. This point represents the best compromise between technical performance and rational use of the adsorbent.

#### 3.2.2 Adsorption Kinetics and Fitted Models

The literature presents consistent evidence regarding the application of kinetic models in adsorption processes with ion-exchange resins. In this context, the adsorption kinetics of acetic acid on ion-exchange resins were investigated using pseudo-first-order and pseudo-second-order kinetic models. The resins reached equilibrium within 60 min, with the pseudo-second-order model being more suitable (adjusted *R* ^2^ > 0.99997).^14^ Similar approaches have also been successfully employed in previous studies.^20,40^

Building upon this foundation, adsorption kinetics were studied to determine the effect of contact time on the process. The results are presented in Figure 5. Consistent with findings reported in the literature, equilibrium was reached at 60 min for the concentration of 20 mg·L^–1^ and at approximately 90 min for the concentration of 50 mg·L^–1^. Notably, the resin presented high initial rates of acetic acid adsorption.

**Figure 5:**
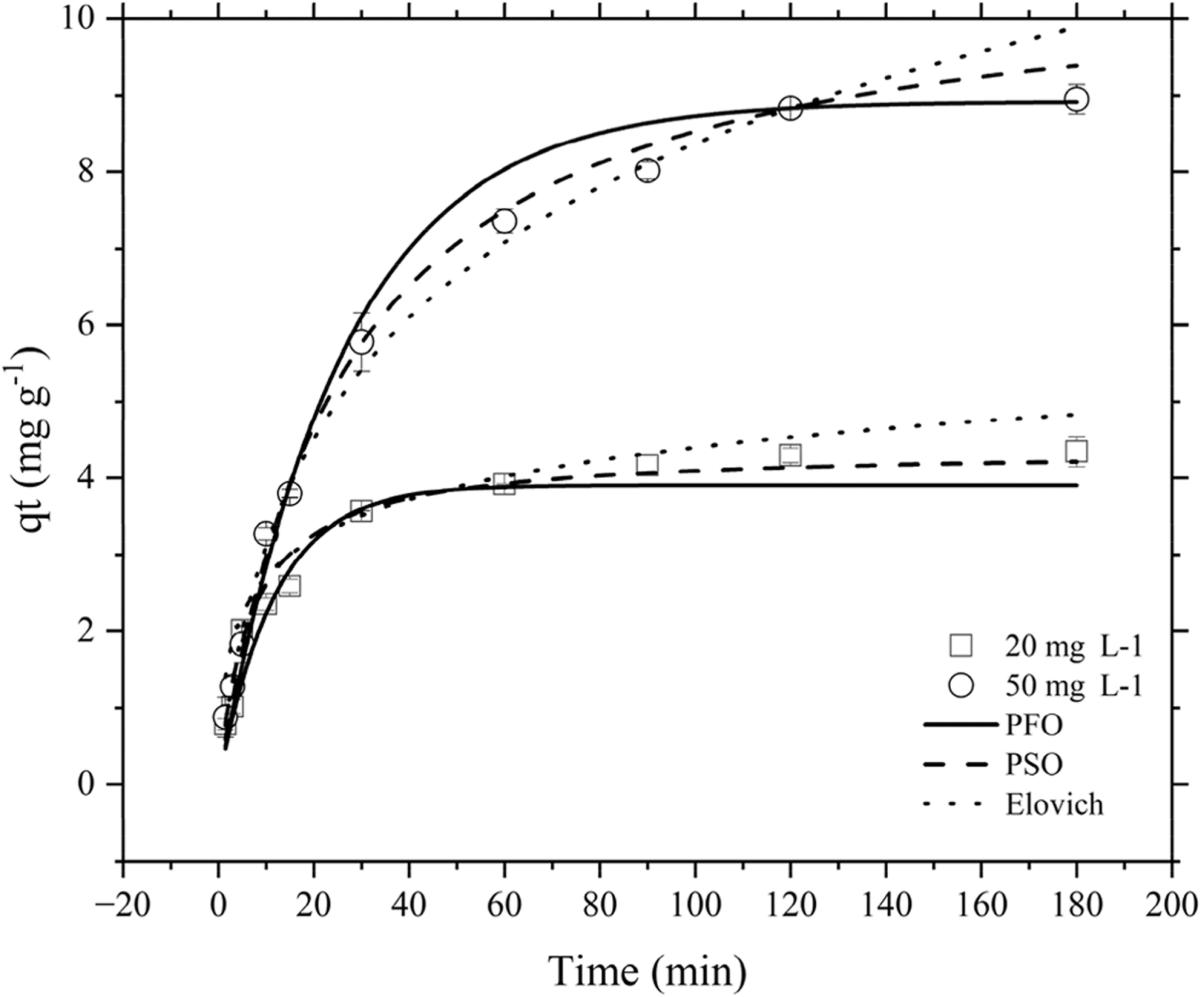
Kinetic curves for acetic acid adsorption on IRA-67 ion-exchange resin (25 °C and adsorbent dosage of 1.389 g·L^–1^).

These equilibrium times align with the previously reported values. Equilibrium for formic acid adsorption onto Amberlite IRA-67 was reached at approximately 90 min,^40^ matching the value obtained for the higher concentration in this work. The variation in the equilibrium time with the initial concentration can be attributed to increased competition for available active sites on the resin surface as more adsorbate molecules are present in solution, resulting in a longer time to reach saturation.

The kinetics of acetic acid adsorption by the ion-exchange resin was evaluated using pseudo-first-order, pseudo-second-order, and Elovich models, and the parameters obtained by these models are presented in Table 1. Among the models tested, the one that best represented the experimental data was the pseudo-second-order model, which presented the highest adjusted *R* ^2^ values, 0.98619 for 20 mg·L^–1^ and 0.99807 for 50 mg·L^–1^, outperforming the pseudo-first-order model (adjusted *R* ^2^ = 0.92838 and 0.99453) and the Elovich model (adjusted *R* ^2^ = 0.90897 and 0.99806). In addition, the pseudo-second-order model exhibited the lowest reduced chi-square values (χ_v_^2^ = 8.19420 and 4.58745) and the lowest absolute relative error (ARE) values (11.29432 and 13.62349), further reinforcing its superiority in representing the adsorption kinetics and indicating a better quality of fit.

**Table 1:** Kinetic Parameters Estimated for Acetic Acid Adsorption on Ion Exchange Resin

		value	
model	parameter	20 mg·L^–1^	50 mg·L^–1^
PFO	*q*_e_	3.90767	8.92468
	*k*	0.08416	0.03833
	reduced Chi-Sqr	42.50092	11.55222
	*R* ^2^	0.93634	0.99513
	*R* ^2^ adjusted	0.92838	0.99453
	ARE	24.92833	37.63399
PSO	*q*_e_	4.37045	10.75403
	*k*	0.03379	0.00357
	reduced Chi-Sqr	8.19420	4.58745
	*R* ^2^	0.98773	0.99807
	*R* ^2^ adjusted	0.98619	0.99807
	ARE	11.29432	13.62349
Elovich	*A*	2.78205	0.59074
	*B*	1.34832	0.37564
	reduced Chi-Sqr	54.02167	4.59911
	*R* ^2^	0.91908	0.99806
	*R* ^2^ adjusted	0.90897	0.99806
	ARE	17.67022	41.89721

These results are in agreement with data reported in the literature for the adsorption of organic acids onto ion exchange resins. The PSO model was found to be superior to the PFO model for acetic acid removal from fuel ethanol using resin 330, with *R* ^2^ = 0.99, compared to *R* ^2^ = 0.94. Similarly, for formic acid adsorption onto Amberlite IRA-67 resin, the PSO model yielded *R* ^2^ = 0.9996, significantly higher than the PFO model (*R* ^2^ = 0.9934).^40^ The consistency of these results across different carboxylic acids and different resin types suggests that the PSO model is the most suitable for describing the adsorption kinetics of these systems. The adsorption capacity calculated by the model (*q*_e_ = 4.37045 and 10.75403 mg·g^–1^) was also consistent with the experimental data, which were 4.34451 and 8.95321 mg·g^–1^.

Good agreement between calculated and experimental *q*_e_ values was observed, particularly for the lower concentration. Good agreement between calculated and experimental *q*_e_ values for the PSO model has also been reported for formic acid adsorption onto ion exchange resins.^40^ The larger discrepancy observed for the 50 mg·L^–1^ concentration (approximately 20% difference between calculated and experimental values) may indicate that the system had not yet fully reached equilibrium or that there are model limitations at higher concentrations.

This suggests that the sorption system does not follow a first-order reaction and that the pseudo-second-order model provides the best correlation to the experimental data. This model is based on the premise that adsorption onto the active site is the dominant step.^41^

A methodological contribution of this work was the use of multiple statistical criteria for the evaluation of kinetic models, including adjusted *R* ^2^, reduced chi-square (χ_v_^2^), and absolute relative error (ARE). The PSO model presented not only the highest adjusted *R* ^2^ values but also the lowest χ_v_^2^ (8.19 and 4.59) and ARE (11.29 and 13.62) values, reinforcing its superiority in representing the experimental data. Unlike previous studies that relied solely on *R* ^2^ for kinetic model comparison,^20,40^ the multicriteria approach adopted in this study allows for a more robust evaluation of fit quality, since *R* ^2^ alone can be misleading, especially when models have different numbers of adjustable parameters.

#### 3.2.3 Equilibrium Studies

The equilibrium isotherms were obtained at 10, 25, and 40 °C from the acetic acid concentrations in the ethanolic solutions after the time required to reach equilibrium. The experimental data were fitted to the Langmuir, Freundlich, and Sips models in order to evaluate the adsorption mechanism, the degree of surface heterogeneity of the resin, and its maximum adsorption capacity.

The experimental curves and model fits are illustrated in Figure 6, while the parameters obtained through regression, including average relative error (ARE), reduced chi-square (χ_υ_^2^), coefficient of determination (*R* ^2^), and adjusted *R* ^2^, are organized in Table 2. The evaluation of model adequacy was performed based on adjusted coefficients of determination (*R* ^2^), reduced chi-square (χ_υ_^2^), and average relative error (ARE), following a widely accepted methodology in the literature where the use of adjusted *R* ^2^ is particularly important in this analysis, as this parameter takes into account the number of variables in each model, allowing for fair comparison between models with different degrees of freedom.^42^

**Figure 6:**
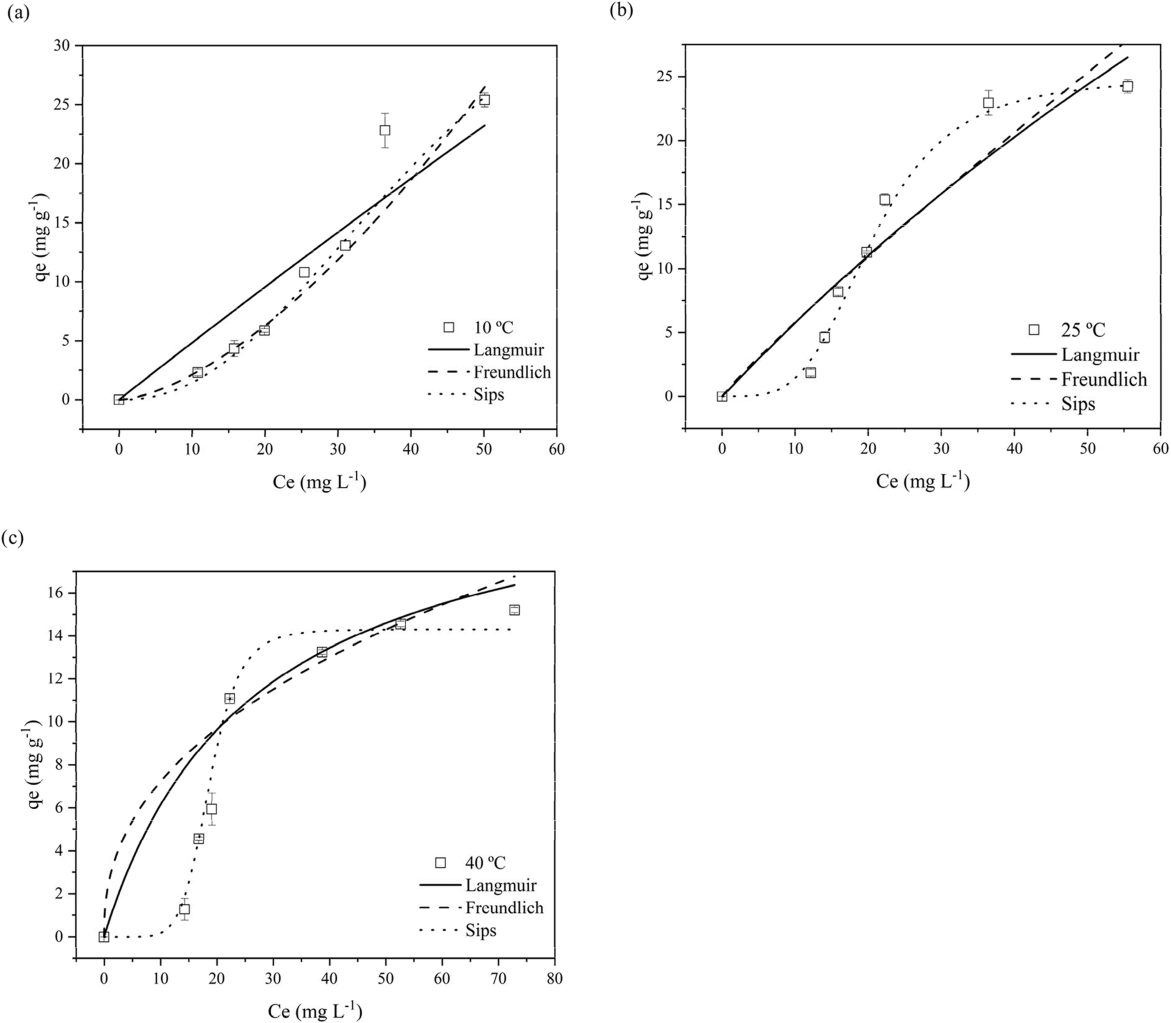
Isotherm curves for the acetic acid adsorption on IRA-67 resin: 10 °C (a), 25 °C (b), 45 °C (c) (adsorbent dosage of 1.389 g·L^–1^ and 2 h).

**Table 2:** Estimated Isothermal Parameters for the Adsorption of Acetic Acid on Ion Exchange Resin

	temperature (°C)		
isotherm model	10	25	40
Langmuir			
*q*_max_	487.2726	127.5540	22.2888
*K*_L_	0.0010	0.0047	0.0380
χ_υ_^2^	128.6305	93.8930	644.5144
*R* ^2^	0.8618	0.9801	0.9758
adjusted *R* ^2^	0.8388	0.9768	0.9718
ARE	34.5409	80.3718	76.3031
Freundlich			
*K*_F_	0.0574	0.7040	2.7211
*n*	0.6380	1.0920	2.3582
χ_υ_^2^	8.3568	100.6911	761.2907
*R* ^2^	0.9910	0.9786	0.9714
adjusted *R* ^2^	0.9896	0.9751	0.9667
ARE	8.8712	80.4991	82.0185
Sips			
*q*_max_	47.4828	24.8701	14.3131
*K*_S_	1.76 × 10^–04^	9.78 × 10^–06^	1.08 × 10^–09^
*n*	0.4445	0.2628	0.1419
χ_υ_^2^	6.3890	11.7405	30.5147
*R* ^2^	0.9943	0.9979	0.9991
adjusted *R* ^2^	0.9920	0.9971	0.9987
ARE	10.2629	31.4694	11.0579

Among the models evaluated, the Sips isotherm showed the best overall performance in representing the adsorption equilibrium. This model, also known as Langmuir–Freundlich, combines the finite-saturation assumption of the Langmuir model with the flexibility of the Freundlich function to describe heterogeneous surfaces. This characteristic allowed it to simultaneously capture the initial heterogeneous behavior and the emergence of a saturation plateau at high concentrations, exactly the pattern observed experimentally.^43^ The *q*_max_ values estimated by the Sips model were physically consistent with the experimental data, ranging from 14 to 25 mg·g^–1^ across the three temperatures studied. In addition, the model exhibited the lowest χ^2^ and ARE values and an adjusted *R* ^2^ above 0.9920, demonstrating excellent agreement with the data.

The superior performance of the Sips model observed in this study presents an interesting contrast with results reported in the literature for carboxylic acid adsorption onto weakly basic anion exchange resins. In aqueous systems, the Langmuir model consistently provides excellent fits: *R* ^2^ > 0.99 was obtained for formic acid adsorption onto Amberlite IRA-67 across all temperatures studied (298–328 K),^40^ while for tartaric and propionic acids on the same resin, Langmuir also shows superior correlation (*R* ^2^ = 0.959–0.999) compared to Freundlich (*R* ^2^ = 0.937–0.990).^44^ Similarly, the Langmuir–Freundlich model (also referred to as the Sips isotherm) provides the best fit (*R* ^2^ > 0.94) for lactic acid adsorption onto Amberlite IRA-67 from aqueous fermentation broth, outperforming the Langmuir model (*R* ^2^ > 0.91).^45^ In the present work, the failure of the Langmuir model, which yields physically unrealistic *q*_max_ values up to 20 times higher than those experimentally observed, suggests that specific characteristics of the system studied, including the concentration range, the acrylic gel matrix of IRA-67, and the potential presence of residual water in the ethanolic medium, introduce substantial surface heterogeneity that only the Sips model is able to capture adequately.

The *n* parameter, lower than 1 at all temperatures (0.44, 0.26, 0.14), indicates a highly heterogeneous surface, with increasing energetic dispersion at higher temperatures. This trend suggests a decrease in acid–resin affinity in ethanolic medium and an intensification of solvation effects as temperature increases, in addition to aligning with the typically exothermic behavior of adsorption systems involving electrostatic interactions.^46^

The Langmuir model showed the poorest performance among the three evaluated models. Although theoretically grounded in monolayer adsorption on a homogeneous surface, its assumptions did not align with the system studied. The model exhibited high coefficients of determination under some conditions but generated physically unrealistic *q*_max_ values, such as 487.27 mg·g^–1^ at 10 °C and 127.55 mg·g^–1^ at 25 °C, far above the experimental values, which ranged from 20 to 25 mg·g^–1^. Moreover, the high χ^2^ and ARE values reveal that the model does not follow the experimental curvature of the isotherm, indicating that the assumptions of uniform adsorption sites and absence of lateral interactions between adsorbed molecules do not adequately represent the behavior of IRA-67 resin in ethanolic medium.^47^

Finally, the Freundlich model, although also capable of capturing surface heterogeneity, showed limitations in describing the saturation regime. At 10 °C, the fitting was satisfactory, with low χ^2^, low ARE, and a high *R* ^2^ (0.9910), reflecting a good representation of the initial curvature of the isotherm. The *n* value of 0.6380 reinforces the presence of an energetically nonuniform surface. However, because it is a strictly empirical model that does not include a maximum capacity term, its performance deteriorated drastically at 25 and 40 °C. Under these conditions, χ^2^ values increased sharply (100.69 and 761.29), accompanied by ARE values above 80%, in addition to their inability to represent the experimentally observed plateau. This limitation is inherent to the model, which describes the initial region well but does not adequately reproduce the behavior at high concentrations.^48^

In summary, the combined evaluation of statistical indicators, the physical plausibility of the estimated parameters, and the visual quality of the model fits shows that the Sips isotherm offers the most robust description of acetic acid adsorption equilibrium onto the IRA-67 resin in an ethanolic medium. The adequacy of this model reflects the intrinsic complexity of the system, in which the acrylic gel matrix and temperature-dependent solvation phenomena lead to a heterogeneous distribution of adsorption site energies that cannot be satisfactorily described by either the Langmuir or the Freundlich models. The gradual decrease in the heterogeneity parameter *n* with an increase in temperature further reinforces this interpretation, as it suggests stronger competition between ethanol and acetic acid molecules for the available active sites. Overall, these results emphasize that adsorption mechanisms established in aqueous systems cannot be directly transferred to ethanolic media without careful reassessment of the equilibrium behavior.

#### 3.2.4 Thermodynamic Parameter Estimates

The thermodynamic analysis of the adsorption process was conducted through the calculation of the Gibbs free energy (Δ*G*), enthalpy (Δ*H*), and entropy (Δ*S*) parameters using Van’t Hoff relations, a methodology widely reported in the literature.^23^ These parameters were obtained from the slope and intercept of the linear relationship between ln *K* and 1/*T*, and the corresponding values are summarized in Table 3. Negative Δ*G* values were observed at all evaluated temperatures (−16.8561, −19.7610, and −21.0195 kJ·mol^–1^ at 10, 25, and 40 °C, respectively), confirming that the adsorption process is spontaneous over the entire temperature range, with increased spontaneity at higher temperatures.^24–26^

**Table 3:** Thermodynamic Properties for Acetic Acid onto IRA-67 Resin

*T* (K)	Δ*G* (kJ·mol^–1^)	Δ*H* (kJ·mol^–1^)	Δ*S* (J·mol^–1^·K^–1^)
283.15	–16.8561	22.71428	140.63081
298.15	–19.7610		
313.15	–21.0195		

The positive enthalpy change (Δ*H* = +22.71 kJ·mol^–1^) indicates that the overall adsorption process is endothermic. This result requires careful interpretation, as the experimental isotherms showed decreased adsorption capacity at higher temperatures, a behavior typically associated with exothermic processes. This apparent contradiction can be reconciled by recognizing that the measured Δ*H* represents the net enthalpy balance of multiple concurrent phenomena in the ethanolic medium, rather than the intrinsic acid–resin interaction alone.^27–30^

In ethanolic systems, adsorption involves several energy-consuming steps: disruption of the acetic acid solvation shell, displacement of ethanol molecules previously associated with the resin surface, and reorganization of the solvent structure around the adsorption site. Another study demonstrated that acetic acid in ethanol presents an activity coefficient close to unity, reflecting strong solvation that must be overcome for adsorption to occur.^39^ Furthermore, these authors reported that ethanol, due to its capacity to act as both a hydrogen bond donor and acceptor, is coadsorbed onto weakly basic resins at nearly twice the level observed for ethyl acetate. Consequently, the energy required to disrupt acid–ethanol and ethanol–resin interactions during adsorption exceeds the energy released upon acid–resin binding, resulting in a net positive enthalpy change.

The markedly positive entropy change (Δ*S* = +140.6 J·mol^–1^·K^–1^) constitutes the main thermodynamic driving force for the spontaneous adsorption observed in this system. This substantial increase in entropy is attributed to the release of ethanol molecules from both the acetic acid solvation shell and the resin surface during adsorption, which significantly enhances the disorder of the system. As stated elsewhere, sorption processes may be either enthalpy-driven or entropy-driven depending on the nature of the sorbate–sorbent–solvent interactions, with hydrophobic bonding representing a classical example of an entropy-driven mechanism in which solvent displacement increases disorder and thermodynamically favors adsorption.^49,50^ In accordance with the Gibbs–Helmholtz equation (Δ*G* = Δ*H* – *T*Δ*S*), the entropic contribution (*T*Δ*S* = 41.9 kJ·mol^–1^ at 25 °C) exceeds the enthalpic penalty (Δ*H* = 22.7 kJ·mol^–1^), rendering the process thermodynamically favorable despite its endothermic character.

The apparent inconsistency between the endothermic nature of the adsorption process (Δ*H* > 0) and the reduction in adsorption capacity observed at higher temperatures in the isotherm experiments can be explained by the competitive role of ethanol. As demonstrated elsewhere, ethanol competes with acetic acid for interaction with the resin’s amine groups through hydrogen bonding.^39^ At elevated temperatures, increased molecular mobility and weakened hydrogen bond stability may intensify this competition, promoting greater ethanol coadsorption and, consequently, a reduced apparent adsorption capacity for acetic acid, even though the intrinsic thermodynamic driving force for acid adsorption (Δ*G*) becomes more favorable. This interpretation is further supported by the progressive decrease in the Sips heterogeneity parameter (*n*) with increasing temperature (from 0.44 at 10 °C to 0.14 at 40 °C), which indicates enhanced site competition and greater energetic heterogeneity among adsorption sites at higher temperatures.

#### 3.2.5 Effect of Competitive Organic Acids

The competitive adsorption results, discussed in detail in the , showed that IRA-67 resin retained good acetic acid removal performance even in the presence of other organic acids commonly found in fuel ethanol. Among the acids tested, butyric acid caused the most significant interference, reducing the removal efficiency from 62 to 47%, likely due to its higher hydrophobicity and molecular volume. Formic and propionic acids had a minimal impact, with removal rates remaining above 60%. These findings suggest that the resin is selective enough for practical applications in complex ethanol matrices.

#### 3.2.6 Regeneration

The regeneration performance of IRA-67 resin over five consecutive adsorption–desorption cycles is discussed in detail in the . In summary, the resin demonstrated good stability throughout the reuse cycles, retaining approximately 85% of its original adsorption capacity after five cycles. The gradual decline observed from the third cycle onward may be associated with partial saturation of active sites or structural changes during repeated regeneration. These results confirm that 3% NaOH solution was effective as a regenerant, supporting the practical feasibility of resin reuse in continuous applications.

#### 3.2.7 Adsorption Mechanism

The structural characterization of the Amberlite IRA-67 resin by Fourier-transform infrared spectroscopy (FT-IR), as can be seen in Figure 7, provides a comprehensive elucidation of the functional groups present in the polymeric matrix and, above all, supports the understanding of the molecular modifications associated with the adsorption of organic acids. In the spectrum of the virgin resin, characteristic bands are clearly observed that confirm the presence of the expected functional groups, including O–H stretching vibrations (3447–3425 cm^–1^) related to hydroxyl groups in the matrix and adsorbed water, aliphatic C–H stretching from the acrylic backbone (2949–2720 cm^–1^), residual carbonyl groups (1652 cm^–1^), and, most notably, the C–N stretching bands corresponding to tertiary amines, the functional group responsible for ion exchange, located between 1305 and 1100 cm^–1^. The agreement between these bands and those reported in the literature reinforces the structural integrity of the resin and confirms the effective presence of the amine groups that play a central role in adsorption.^40,44,51^

**Figure 7:**
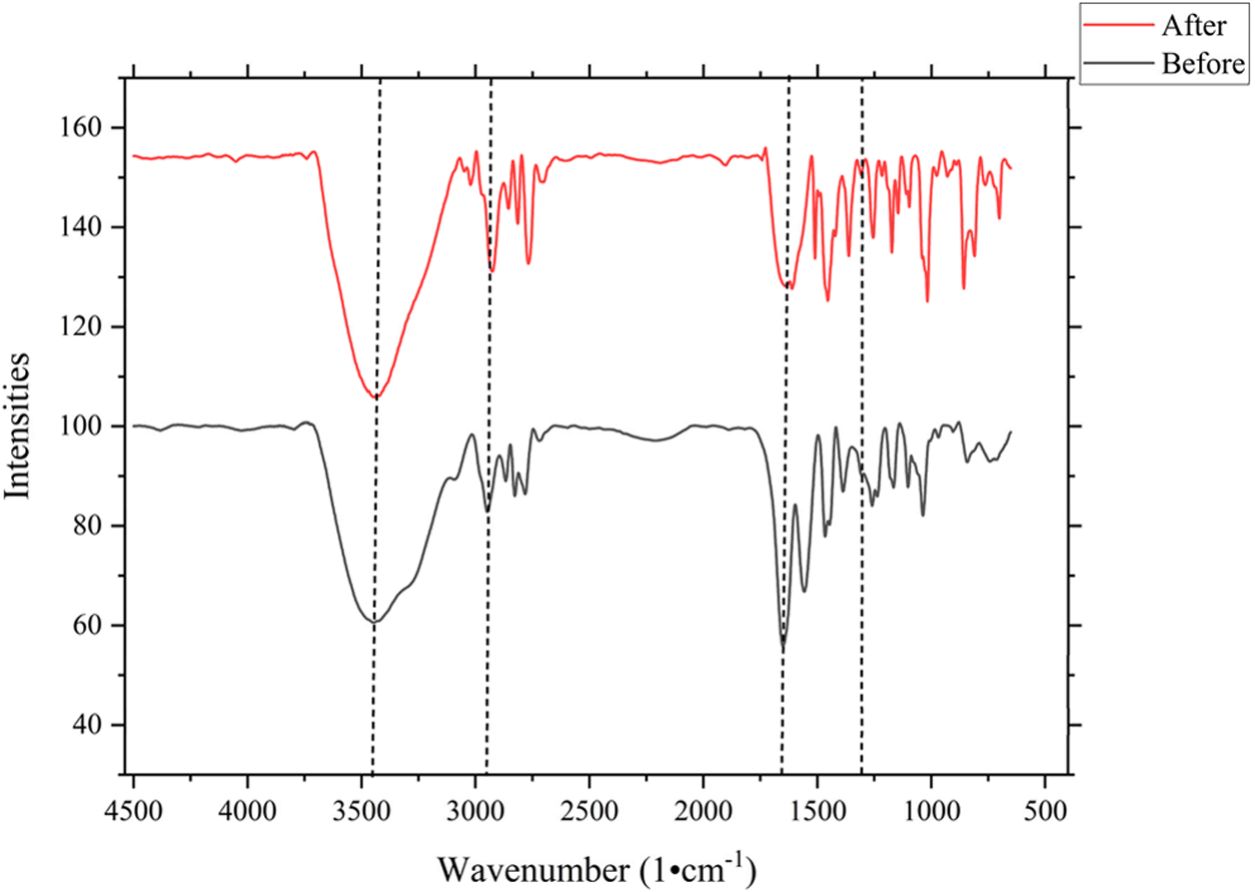
FT-IR spectra of Amberlite IRA- 67 resin before and after utilization.

After the adsorption process, the FT-IR spectrum exhibits systematic changes that directly reflect the interaction between the resin and the organic acids. The shift and broadening of the O–H band to 3472–3445 cm^–1^ suggest the strengthening of hydrogen bonding between the carboxylic groups of the acids and the hydroxyl and nitrogen groups of the resin. This behavior is consistent with what has been reported in the literature,^40^ who observed similar shifts during the adsorption of formic acid. In that work, the −OH/–NH_2_ band shifted from 3275 to 3330 cm^–1^, while the C=O stretching shifted from 1635 to 1645 cm^–1^ after adsorption. These shifts to higher wavenumbers are attributed to hydrogen bond formation between the amine groups and the carboxyl group of the acid. The similar behavior observed in the present ethanolic system confirms that hydrogen bonding plays a central role in both aqueous and nonaqueous media.

At the same time, the region corresponding to C–H stretching remains essentially unchanged, indicating that the acrylic structure does not undergo degradation during adsorption. In contrast, the C–N stretching bands exhibit subtle shifts, consistent with the partial protonation of tertiary amines and the formation of ion pairs. Although slight, these changes show that the electronic environment around the nitrogen is significantly influenced by the acid–base interaction, without indicating complete protonation of the resin, a phenomenon observed only under intensive acid treatment. Thus, the spectra indicate that the resin acts predominantly in its free-base form, being protonated only in situ during contact with the acids.

The comparison with literature data reinforces this interpretation. Substantial spectral changes from HCl conditioning, including more pronounced shifts in the C–N bands and the appearance of peaks typical of fully protonated amines, were not observed in this study, in contrast to previously reported findings.^51^ This demonstrates that the adsorption process occurs under mild conditions and does not cause structural damage to the resin or permanent alterations to its functional groups. In parallel, the stability of the C–H and C=O bands confirms the robustness of the acrylic matrix, an essential characteristic for applications involving successive adsorption and regeneration cycles.

The integrated analysis of the spectral modifications allows the nature of the interactions between the resin and the adsorbate to be clearly identified. The results indicate that the process is dominated by chemisorption rather than by mechanisms governed solely by low-energy physical forces. The central mechanism involves ion exchange, in which proton transfer from the acid to the tertiary amine occurs, forming the ionic complex. The observed changes in the C–N bands and the characteristic selectivity of weak-base resins reinforce this understanding. A detailed mechanistic interpretation for acetic acid adsorption onto weakly basic resins in nonaqueous media is provided elsewhere.^39^ In organic solvents, where the low dielectric constant minimizes acid dissociation, classical ion-exchange becomes negligible. Instead, the dominant mechanism involves hydrogen-bonded complex formation between the acidic proton and the lone pair of electrons on the amine nitrogen, a Lewis acid–base interaction. The authors quantified this using solvatochromic parameters: acetic acid acts as a Lewis acid, while the tertiary amine acts as a Lewis base, explaining the preferential acid–resin complexation. The energy associated with this type of interaction is substantially greater than that of van der Waals forces, highlighting its chemical nature.

The chemisorptive nature inferred from spectroscopic evidence is characteristic of adsorption processes involving specific chemical interactions rather than nonspecific physical forces. The magnitude of the spectral shifts observed, particularly the displacement of the O–H band and the subtle changes in C–N stretching, indicates the formation of hydrogen bonds and modification of the electronic environment around the amine nitrogen, consistent with Lewis acid–base complex formation.^39^ These spectroscopic signatures are incompatible with physisorption mechanisms, which typically produce minimal or no changes in the infrared spectrum of the adsorbent. Furthermore, the shift in the O–H band indicates an additional contribution from hydrogen bonds, which help stabilize the complexes formed. van der Waals interactions, although present as background forces, play a secondary role and do not govern the process.

The superior performance of weakly basic resins compared with strongly basic resins for carboxylic acid adsorption from organic media has a structural explanation. Weakly basic resins exhibited higher capacity for acetic acid removal from ethanol compared to strongly basic resins.^20^ This difference can be attributed to the accessibility of the nitrogen lone pair: in quaternary ammonium groups, steric hindrance prevents effective hydrogen-bond formation, while tertiary amines readily participate in hydrogen bonding with the acidic proton.^39^ Additionally, the moderate interaction strength of tertiary amines allows effective regeneration without structural damage to the resin, as confirmed by FT-IR analysis in the present study.

The distinction between chemisorption and physisorption becomes clear when considering simultaneously the spectral shifts, the modifications to the electronic environment of the amines, the formation of specific chemical bonds, the need for chemical regeneration to release the adsorbate, and the structural preservation of the resin. These characteristics are not compatible with mechanisms governed exclusively by physisorption, which are marked by weak interactions and an immediate reversibility.

The medium containing ethanol introduces competitive effects that influence the adsorption mechanism. As ethanol is capable of acting as both a hydrogen bond donor and acceptor, it competes with acetic acid for the resin’s amine groups.^39^ Their measurements showed ethanol coadsorption at nearly twice the level observed for ethyl acetate. This competition reduces overall adsorption capacity and contributes to surface heterogeneity, which is consistent with the superior fit of the Sips isotherm model (*n* < 1) observed in the equilibrium studies of the present work.

On the basis of these results, an integrated adsorption mechanism can be proposed in which the acid initially approaches the resin surface by diffusion, establishes hydrogen bonds between the carboxylic group and the nitrogen of the amine, and subsequently undergoes proton transfer that leads to the formation of a stable ion pair. This model aligns with both the spectroscopic evidence obtained and previous reports in the literature, contributing to a more comprehensive understanding of the adsorption of organic acids by Amberlite IRA-67 resin.

The adsorption of acetic acid onto Amberlite IRA-67 in ethanolic medium involves multiple concurrent mechanisms: hydrogen bonding between carboxylic and amine groups, partial proton transfer forming ion pairs, and competitive ethanol adsorption. The FT-IR evidence obtained in this study, the thermodynamic data from the literature, and the excellent fit of the Sips isotherm model collectively support this multimechanism interpretation. Unlike aqueous systems, where classical ion-exchange dominates due to extensive acid dissociation, the ethanolic medium favors hydrogen bonding and Lewis acid–base interactions as the primary adsorption mechanisms, while maintaining the chemical selectivity characteristic of weakly basic anion exchange resins.^20,39^

#### 3.2.8 Removal of Acetic Acid from Ethanol Fuel Samples

The assay performed with real ethanol samples from different refineries and fuel stations demonstrated a significant reduction in acetic acid concentration after adsorption with IRA-67 resin. Table 4 presents the initial and final acetic acid values for each sample as well as the respective removal efficiency.

**Table 4:** Acid Concentration and Adsorption Percentage with IRA-67 Resin from Ethanol Fuel Samples

	acetic acid concentration		
samples	before (mg·L^–1^)	after (mg·L^–1^)	acetic acid removal (%)
1	35,268	20,981	41
2	7453	5202	30
3	36,165	21,973	39

The results obtained with real fuel ethanol samples clearly demonstrate that IRA-67 resin effectively removes acetic acid under practical conditions, achieving efficiencies that range from 30 to 41%. This variation reveals how much more complex commercial matrices are compared to the controlled synthetic solutions prepared in the laboratory. The notable difference in initial acetic acid concentrations across samples (7.4–36.2 mg·L^–1^) highlights the inconsistent quality of fuel ethanol available in the marketplace, which likely stems from varying production processes, storage conditions, or different suppliers. Interestingly, samples 1 and 3 from the states of MS and MT, respectively, which showed similar and relatively high initial concentrations of acetic acid, delivered comparable removal efficiencies (41 and 39%, respectively), indicating that the resin performs consistently when dealing with higher concentration levels.

The lower efficiency observed in removing acetic acid from sample 2 from the state of GO (30%) is likely due to the reduced driving force for mass transfer at low initial concentrations. In adsorption processes, the concentration gradient between the solution and the adsorbent serves as the primary driving force, and when this gradient is diminished, it hinders the diffusion of the adsorbate to the active sites, creating kinetic limitations that result in lower adsorption rates and capacities.^52^ This effect is compounded by interference from other compounds naturally present in the commercial ethanol matrix, which compete for adsorption sites with acetic acid, as demonstrated in Section 3.2.5.

From an industrial and regulatory standpoint, the removal efficiencies between 30 and 41% obtained in a single-stage batch process have relevant practical implications. According to ANP Resolution No. 907 of 2022, the maximum allowable acidity in fuel ethanol is 30 mg·L^–1^.^31^ In this context, samples 1 and 3 exhibited initial acetic acid concentrations of 35.3 and 36.2 mg·L^–1^, respectively, values that exceeded the regulatory limit and would therefore classify these fuels as noncompliant for commercial distribution. After a single adsorption stage using IRA-67 resin, the acetic acid concentrations were reduced to 21.0 and 22.0 mg·L^–1^, respectively, bringing both samples into full compliance with ANP specifications. These results demonstrate that the ion exchange treatment was able to convert noncompliant fuel ethanol into a product suitable for commercialization through a single processing step, which represents a significant advantage from an industrial implementation perspective.

For samples with higher contamination levels or for applications that demand stricter purity requirements, configurations involving multiple stages can be adopted. On the basis of the removal efficiencies observed, a two-stage sequential batch process would theoretically result in overall removals between 51 and 65%, while a three-stage system could achieve removals in the range of 66–79%. Alternatively, the fixed-bed column results discussed in Section 3.2.9 indicate that continuous flow operation provides initial removal efficiencies above 95%, suggesting that column systems may offer superior performance for industrial applications when compared to batch processes. The selection between batch and continuous operation would depend on factors such as plant throughput, capital investment constraints, and variability in the feedstock quality. Nonetheless, the demonstrated ability of the IRA-67 resin to reduce acetic acid concentrations in noncompliant ethanol to levels within regulatory limits in a single stage, together with its satisfactory regenerability, maintaining approximately 85% efficiency after five cycles, provides strong evidence supporting the technical and economic feasibility of ion exchange technology for fuel ethanol purification at an industrial scale.

Although corrosion experiments after acid removal were not performed in this study, the results obtained in the corrosion assays (Section 3.1) clearly demonstrated that acetic acid concentration is a key driver of corrosion in fuel ethanol systems. Given that IRA-67 resin was able to reduce acetic acid concentrations to levels below the regulatory limit of 30 mg·L^–1^ established by ANP Resolution No. 907 of 2022, it is expected that such a reduction would contribute to a corresponding mitigation of corrosive activity in fuel system components. Future studies should directly evaluate the corrosion behavior of metallic components exposed to treated ethanol to experimentally validate this hypothesis.

#### 3.2.9 Breakthrough Curves

Figure 8 shows the behavior of the acetic acid concentration in the effluent and the removal efficiency over time. In the first 5 min of operation, the concentration in ethanol is close to zero, which demonstrates high adsorption efficiency, a direct result of the great availability of active sites in the resin. In this initial stage, removal remains close to 95%.

**Figure 8:**
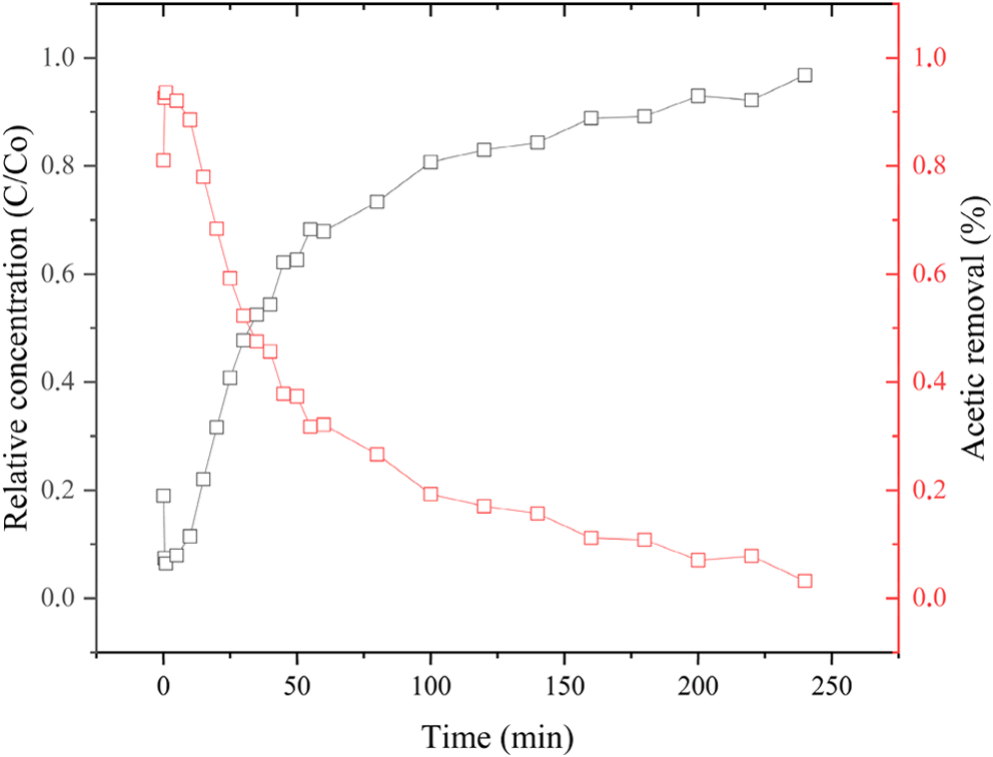
IRA-67 resin breakthrough curve.

As time progresses, the resin exhaustion phase begins, noticeable by the gradual increase in the acetic acid concentration in the effluent, accompanied by a drop in removal efficiency. Around 50 min, the curve begins to stabilize, suggesting that most of the adsorptive sites are being occupied. At the end of the test, after 240 min of operation, the concentration approaches the initial value, indicating that the maximum adsorption capacity of the column was practically reached. This finding aligns with previous research that demonstrated the column’s ability to capture nearly 100% of the acetic acid from the influent in the first minutes, showing the efficiency of the system.^14^

This profile is characteristic of fixed-bed systems, in which the mass transfer zone moves along the column until reaching saturation.^53^ Understanding this behavior is essential for the correct dimensioning of continuous systems, allowing prediction of optimal operation time before it becomes necessary to regenerate or replace the adsorbent material.

## 4 Conclusion

The results obtained in this study clearly demonstrate the critical role of organic acids, particularly acetic acid, in the corrosivity of fuel ethanol on metallic materials widely used in automotive injection systems. Prolonged exposure of stainless-steel injector valves to different ethanol samples revealed that increased acidity, especially from short-chain acids such as acetic and formic acids, is directly associated with the intensification of the corrosive process.

The evaluation of acetic acid removal using ion-exchange resins demonstrated that Amberlite IRA-67, functionalized with tertiary amine groups, showed the best performance among the tested materials, both in laboratory-prepared solutions and in real commercial ethanol samples. The efficiency of the adsorption process was confirmed through kinetic, equilibrium, and thermodynamic studies, with the pseudo-second-order model and Sips isotherm providing the best description of the experimental data. Thermodynamic analysis revealed an entropy-driven process, where solvent release provides the main driving force for spontaneous adsorption, despite the endothermic enthalpy change. The resin also proved to be effective even in the presence of other organic acids, experiencing only moderate reductions in capacity, while demonstrating good regenerability with significant maintenance of efficiency after multiple reuse cycles.

Tests conducted in a fixed-bed system operating under continuous flow reinforced the potential of this technology as a viable alternative for industrial-scale applications, demonstrating a high initial removal efficiency and characteristic behavior of materials with well-defined mass transfer zones. Notably, the treatment was capable of reducing acetic acid concentrations in noncompliant fuel ethanol samples to levels within ANP regulatory limits through a single processing stage.

Thus, this work confirms the technical feasibility of applying ion-exchange resins in fuel ethanol purification, directly contributing to the reduction of corrosive effects and the improvement of biofuel quality. The presented results provide relevant support for the development of industrial strategies aimed at protecting metallic components and promoting greater system durability and safety in the use of ethanol as a fuel.

## Supplementary Material





## Notes

Materials and methods; corrosion assays; fuel ethanol sample; synthetic ethanol sample; adsorption with ion exchange resin; analytical methods (DOCX)
Results and discussion; adsorption tests; removal capacity and efficiency in the reusability of the IRA-67 resin (DOCX)
