# Supplementary material for: Self-Induced Acidification of Fuel Ethanol and Its Role in Corrosion: Mitigation via Ion-Exchange Resins
Source: ACS Omega. 2026 Jun 26;11(27):40511–25. doi: 10.1021/acsomega.6c03466 (PMC13382688; doi:10.1021/acsomega.6c03466)
Supplement: Supplementary file 1 [file ao6c03466_si_001.docx]

# S.1 Materials and methods

## S1.1 Materials

Absolute anhydrous ethyl alcohol 99.8% P.A. was purchased from Êxodo Científica, acetic acid 99.8% P.A was purchased from NEON (Brazil), butyric acid 99% and propionic acid 99.5% were purchased from Merck Co., and formic acid 85% P.A. was purchased from ACS Científica. All reagents used in this study were of analytical grade. Stainless steel fuel injector valves were donated by an automotive industry company. The adsorbents used in this study were commercial ion exchange resins. The IRA-67 and IRA-96 resins were purchased from Merck Co. Some physicochemical properties of the resins are presented in Table S1.

**Table S1.** Basic properties of the resins.

| Property | IRA 67 | IRA 96 |
| --- | --- | --- |
| Copolymer | Crosslinked acrylic | Styrene-divinylbenzene |
| Matrix | Gel | Macroporous |
| Type | Weak base anion | Weak base anion |
| Functional Group | Tertiary amine | Polyamine |
| Physical Form | White, translucent beads | White to beige, opaque beads |
| Ionic Form (as supplied) | Free base (FB) | Free base (FB) |
| Total Exchange Capacity | ≥ 1.60 eq L^-1^ (free base form) | ≥ 1.3 eq L^-1^ (free base form) |
| Water Retention Capacity | 56.0 – 62.0% (free base form) | 59.0 – 65.0% (free base form) |
| Particle Diameter | 500 – 750 µm | 550 – 750 µm |
| Uniformity Coefficient | ≤ 1.80 | ≤ 1.60 |
| Particles < 300 µm | ≤ 1.0% | ≤ 1.0% |
| Particles > 1180 µm | ≤ 5.0% | ≤ 5.0% |
| Swelling (FB → HCl) | ≥ 20% | 15% |
| Particle Density | 1.07 g mL^-1^ | 1.06 g mL^-1^ |
| Temperature Range (FB) | 5 – 60 °C | 5 – 60 °C |
| pH Range (Service Cycle) | 0 – 6 | 0 – 6 |
| pH Range (Stable) | 0 – 14 | 0 – 14 |

Source: Manufacturer's technical datasheet Merk co., 2025.

In addition to the synthetic (laboratory-prepared) solutions, real samples of fuel ethanol were also used, collected from different fuel stations and refineries in the central and southern regions of Brazil, to evaluate the corrosion resistance of stainless steel fuel injector valves under conditions normally found.

For the analysis with synthetic ethanol, the solution was prepared using anhydrous ethyl alcohol P.A., to which Milli-Q water was added until reaching a concentration of 5% vol. water, characterizing hydrated ethanol (95% v/v).

## S1.2 Corrosion Assays

The present immersion assays were developed based on previous studies that employed distinct methodologies for corrosion evaluation in fuel ethanol. One approach involved 30-day immersion in sealed tubes containing stainless steel valves at room temperature, with subsequent morphological analysis of surfaces ^12^. Another approach conducted 8-day exposure assay in glass flasks with controlled atmosphere by gas purge system, evaluating mass loss, density, and pit size, in addition to performing characterizations by optical and electron microscopy ^18^. The methodology adopted here followed the same general principles, but with specific adaptations regarding immersion time, sample type, and analytical procedures, as described in the following subsection.

Corrosion assays were conducted with the objective of evaluating the integrity of stainless steel fuel injector valves immersed in different ethanol samples. These assays were conducted in an experimental apparatus constructed based on the guidelines of ASTM G31-21, (2021) standard (Figure 1). The system consisted of a round-bottom volumetric flask containing the ethanolic solution (1), heated by an electric heating mantle adjusted to maintain the temperature at 70 ± 3 °C (2). To prevent evaporation losses, a vertical condenser (3) was coupled to the flask, connected to an ultra-thermostatic bath (4). The metallic samples (5) were fixed by nylon threads (6) and carefully suspended inside the flask, ensuring total immersion in the solution throughout the entire experiment. The entire assembly was supported by a universal stand (7), ensuring stability during continuous operation.


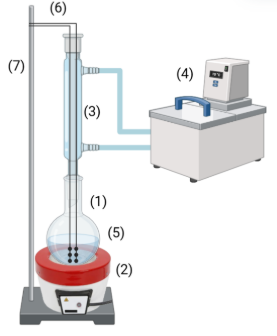

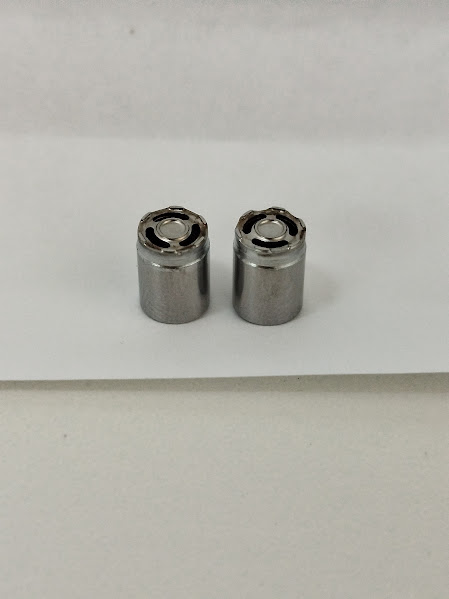


Figure S1. Experimental apparatus of corrosion test of stainless steel fuel injector valves.

### S1.2.1 Fuel Ethanol Sample

Initially, corn ethanol samples were used, in both anhydrous and hydrated forms, collected directly from a distillery in the central-west region of Brazil. The stainless steel fuel injector valves were immersed for 90 days, and ethanol samples were collected at the initial time and after the immersion period for subsequent analysis, to monitor possible changes in the ethanol composition.

In a second stage, a new assay was performed using three commercial ethanol samples: two from corn origin (obtained from a corn ethanol distillery) and one from a fuel station, with no confirmation of feedstock origin. Based on the primary assay, it was decided to assess the solutions every 30 days, during 90 total days of exposure to have better control of the organic acids concentration.

### S1.2.2 Synthetic Ethanol Sample

After verifying an increase in acetic acid content in ethanol, synthetic ethanol solutions with acetic acid concentrations of 46.8, 91.3, and 169.8 mg•L⁻¹ were prepared, along with a control solution without acetic acid addition, to evaluate the direct effect of acetic acid on the corrosion process. The solutions were maintained under the same immersion conditions and periodically monitored regarding acid concentration.

## S1.3 Adsorption with Ion Exchange Resin

### S1.3.1 Batch adsorption Experiments

Before using adsorption experiments, all resins were subjected to a conditioning procedure following the supplier's recommendations. The resins were initially washed with distilled water until complete removal of soluble impurities and fine particles. Subsequently, the material was dried in an oven at 60 °C until constant mass, ensuring removal of residual moisture, aiming to ensure reproducibility of results and optimize the adsorptive capacity of the resins.

#### S1.3.1.1 Adsorbent Dosage

The adsorbent dosage was performed using a procedure already established in the literature ^20^, with some methodological adaptations. A dosage study using the ion exchange resins Amberlite IRA-67 and Amberlite IRA-96 was conducted with dosages ranging from 0,5 to 5 g•L^-1^. Different adsorbent dosages were added to 100 mL of an ethanol solution with a concentration of 20 mg•L^-1^ of acetic acid and maintained under constant agitation for 24 h at 25 °C. The adsorption capacity [$q_{t}$ (mg•g^-1^)], the amount adsorbed at equilibrium [q_e_ (mg•g^-1^)], and the removal efficiency [RE (%)] were determined by Equations (1) – (3), respectively.

$q_{t}=\frac{\left( C_{O}-C_{t} \right)*V}{W}$ (S1)

$q_{e}=\frac{\left( C_{O}-C_{e} \right)*V}{W}$ (S2)

$\mathrm{RE}\left( \% \right)=\frac{\left( C_{O}-C_{e} \right)}{C_{O}}*100$ (S3)

where C_0_, C_e_ e C_t_ are the solution concentrations (mg•L^-1^) at the beginning, at equilibrium, and at time t, respectively. W is the mass of adsorbent (g) used, and V is the volume of the solution (L).

#### S1.3.1.2 Adsorption Kinetics

In the kinetic study, the ideal dosage value (1.389 g•L^-1^) obtained in previous experiments was kept fixed. Additionally, the kinetic evaluation was conducted with an initial concentration of 20 mg•L^-1^ and 50 mg•L^-1^ of acetic acid. Samples were collected at specific time intervals during a total adsorption time of 180 minutes. The kinetic data were tested using three different models, such as PFO ^21^, PSO ^22^ e Elovich ^21^, expressed in equations (4) – (6), respectively.

$q_{t}=q_{1}(1-e^{-k_{1}*t})$ (S4)

$q_{t}=\frac{k_{2}tq_{2}^{2}}{1+k_{2}tq_{2}}$ (S5)

$q_{t}=\frac{1}{B} ln(1+ABt)$ (S6)

where k_1_ (min^-1^) e k_2_ (g•mg^-1^•min^-1^) are the kinetic constants of the PFO and PSO models, respectively, q_1_ and q_2_ are the theoretical values of adsorption capacity (mg•g^-1^), B is the initial rate (mg•L^-1^•min^-1^) and A is the desorption constant of the Elovich model (g•mg^-1^).

#### S1.3.1.3 Equilibrium Studies

Adsorption isotherms were obtained using the optimal dosage and kinetic values obtained in previous experiments. This procedure was performed with various acetic acid concentrations ranging from 10 to 100 mg•L^-1^ at three different temperatures (10, 25, and 40 °C). The equilibrium isotherm curves were fitted with three models, such as Langmuir, Freundlich ^23^ and Sips ^24^ represented by Equations (7) – (9), respectively.

$q_{t}=\frac{Q_{\max}*K_{L}*C_{e}}{1+K_{L}*C_{e}}$ (S7)

$q_{t}= K_{F}*\left( \mathrm{Ce} \right)^{\frac{1}{n}}$ (S8)

$q_{t}=\frac{Q_{\max}*K_{S}*C_{e}^{1/n}}{1+K_{S}*C_{e}^{1/n}}$ (S9)

where K_L_ (L•mg), K_F_ (mg•g^-1^·(mg•L^-1^) ^−1/n^) e K_S_ (mg•L^-1^)^-1/n^ are the equilibrium constants of the Langmuir, Freundlich and Sips models respectively, n is the Freundlich and Sips exponent (dimensionless) and Q_max_ is the maximum Sips adsorption (mg•g^-1^).

#### S1.3.1.4 Thermodynamic Parameter Estimates

Thermodynamic parameters provide valuable insights into the nature of adsorption processes. They are frequently used to assess whether the adsorption is exothermic or endothermic, physical or chemical, and whether it occurs spontaneously or not ^20^. The most studied parameters include the standard Gibbs free energy (ΔG⁰, kJ•mol^-1^), enthalpy (ΔH⁰, kJ•mol^-1^), and entropy (ΔS⁰, J•mol^-1^•K^-1^). At a given solution temperature, the sign of ΔG indicates whether the adsorption process occurs spontaneously (ΔG < 0) or non-spontaneously (ΔG > 0). Additionally, the sign of ΔH⁰ helps determine whether the process is exothermic (ΔH⁰ < 0) or endothermic (ΔH⁰ > 0), while the magnitude of this value reveals whether the interaction is likely due to physisorption (≤ 60 kJ•mol^-1^) or chemisorption (≥ 200 kJ•mol^-1^). These parameters were estimated according to Equations (10) and (11) ^25^.

$\Delta G^{\circ}=-RTln(k^{0})$ (S10)

$\ln\left( k^{0} \right)=\frac{\Delta S^{0}}{R}-\frac{\Delta H^{0}}{\mathrm{RT}}$ (S11)

The equilibrium constant $K_{e}^{0}$ applied in these equations corresponds to the constant obtained from the isotherm model that provided the best fit. Because the equilibrium constants possess units it is necessary to convert them into a dimensionless form ^25,26^. For this purpose, Eq. (12) was applied to convert the Sips constants.

$K_{e}^{0}={10}^{3}*\sqrt[\frac{1}{n}]{\mathrm{Ks}}*M_{w}*\frac{C_{\mathrm{Adsorbate}}^{o}}{\gamma_{\mathrm{Adsorbate}}}$ (S12)

#### S1.3.1.5 Effect Competitive Organic Acids

To characterize the competitive behavior of organic acids, an experimental strategy based on protocol reported in the literature ^27^ was employed, adapted to the specificities of the organic acids/ethanol system. Considering that fuel ethanol generally presents a mixture of different acids, it becomes essential to evaluate the competitive adsorption of coexisting anions in acetic acid removal.

Four acids commonly associated with ethanol acidity were selected: acetic, formic, butyric, and propionic acids, and all acids were used at an individual concentration of 100 mg•L⁻¹. Five ethanol solutions (100 mL each) were prepared in the following combinations: (1) 100 mg•L⁻¹ acetic acid (control), (2) 100 mg•L⁻¹ acetic acid + 100 mg·L⁻¹ formic acid, (3) 100 mg•L⁻¹ acetic acid + 100 mg•L⁻¹ butyric acid, (4) 100 mg•L⁻¹ acetic acid + 100 mg•L⁻¹ propionic acid, and (5) 100 mg•L⁻¹ each of acetic, formic, butyric, and propionic acids. The objective was to assess the competitive influence of formic, butyric, and propionic acids on acetic acid adsorption.

The assays were conducted with an adsorbent dosage of 1.389 g•L⁻¹ (value obtained in section 3.2.1), added to 100 mL of each ethanol solution at a temperature of 25 °C and under constant agitation of 150 rpm for 2 h. After the adsorption process, acetic acid concentration was specifically monitored in all solutions to evaluate the competitive interference of coexisting anions.

#### S1.3.1.6 Resin Regeneration

A similar regeneration approach has been documented in prior research, where three consecutive adsorption–desorption cycles were performed to evaluate the effect of resin reuse on adsorption performance. Each cycle, conducted in triplicate, involved adsorption from a 10 g•L^-1^ acetic acid solution with 1.389 g of resin for 1.5 h at 150 rpm. Desorption was then performed with the optimal solvent, shaking for 1 h at 150 rpm. Afterward, the resin was rinsed with deionized water to remove residual solvent and acid ^28^.

Following this established framework, the present study evaluated adsorbent regeneration efficiency as a fundamental requirement for practical application. To verify the possibility of material reuse, regeneration cycles were performed on samples previously saturated with acetic acid. Initially, adsorption was conducted using an ethanol solution with a concentration of 100 mg•L^-1^, under constant agitation of 150 rpm, for 2 h at 25 °C, employing an adsorbent dosage of 1.389 g•L^-1^. After the adsorption process, the resin was treated with 3% NaOH solution, under agitation of 150 rpm at 25 °C for 1 h. Following NaOH treatment, the adsorbent was washed with distilled water and dried in an oven at 60 °C. Subsequently, the adsorbent was reused in a new adsorption assay, with this procedure being repeated for five cycles.

#### S1.3.1.7 Removal of acetic acid from the real fuel ethanol samples

In addition to the synthetic solutions, three assay were also performed using real ethanol samples collected from different refineries and fuel stations in the states of Goiás, Mato Grosso, and Mato Grosso do Sul. Each assay was conducted using 100 mL of 95% hydrated ethanol, to which IRA-67 adsorbent resin was added at a concentration of 1.389 g•L^-1^. The mixtures were subjected to constant agitation for 2 hours at 25 °C and 150 rpm, to evaluate the removal efficiency of contaminants, particularly acetic acid.

### S1.3.2 Column separation

A literature-based procedure was adopted for the column separation step ^29^, with appropriate modifications. Continuous flow adsorption tests were performed using a 316 stainless steel column (1.0 cm internal diameter and 12.5 cm height), operating under constant pressure of 4 MPa. The resin employed was IRA-67, which was packed between layers of glass beads and positioned symmetrically between the column inlet and outlet. The feed solution consisted of ethanol containing acetic acid at a concentration of 200 mg•L^-1^, being pumped to the column at a flow rate of 6.5 mL•min^-1^. The experiments were conducted at 25 ºC. The duration of each assay was 4 hours, with samples collected at the following intervals: 0.1 s, 30 s, 1 min, every 5 min up to 1 h and subsequently every 20 min. The acetic acid concentration in the effluent was determined by gas chromatography (GC).

## S1.4 Analytical Methods

Quantitative analyses were performed by gas chromatography (GC) (Shimadzu, Nexis GC-2030, Tokyo, Japan) equipped with flame ionization detector (FID) and a SH-Wax capillary column (Shimadzu, 30 m x 0.32 mm × 0.25 µm), based on the procedures described in standard NBR 16041 (2015), and the methodology proposed in previous studies ^28^, but with specific adaptations for this study, in order to optimize precision and adequacy to the characteristics of the analyzed samples. The injector temperature was 250 °C and pressure was 66.2 kPa. The column flow was 2.14 mL•min^-1^, with linear velocity of 36.2 cm•s^-1^. **Split** mode was used with a split ratio of 10:1 and total flow of 26.6 mL•min^-1^. The purge flow was maintained at 3.0 mL•min^-1^. The primary pressure was 3.0 kPa. The column temperature was maintained at 55 °C, with an equilibration time of 1.0 min.

To determine the retention time of acetic acid and construct the analytical curves, synthetic standard solutions of acetic acid in ethanol were prepared, with concentrations ranging from 6.5 to 1000 mg•L^-1^, covering the expected range in the analyzed samples. The samples were properly prepared and submitted to analysis, respecting repeatability and quality control criteria. Quantification of acetic acid was obtained by comparing the areas of the chromatographic peaks of the samples with the areas of the calibrated standards, ensuring accuracy and precision of the results.

The IRA-67 resin used in this study was characterized by Fourier-transform infrared spectroscopy (FT-IR) in order to identify the main functional groups present in the material prior to the adsorption experiments. Additionally, FT-IR analysis was employed to support the interpretation of the adsorption mechanism by monitoring changes in characteristic vibrational bands associated with the interaction between acetic acid and the resin’s functional groups. The analyses were carried out using a Shimadzu Prestige 21 instrument, performed in the range of 4500–500 cm⁻¹.

Statistic 9.1 software (Statsoft, USA) was used to calculate kinetic and equilibrium parameters through nonlinear regression utilizing minimization of least square function. Determination coefficient (R^2^), adjusted determination coefficient (R^2^ adj), reduced chi-square ^24^, and average relative error (ARE) were used to quality adjustment.
