# Supplementary material for: Self-Induced Acidification of Fuel Ethanol and Its Role in Corrosion: Mitigation via Ion-Exchange Resins
Source: ACS Omega. 2026 Jun 26;11(27):40511–25. doi: 10.1021/acsomega.6c03466 (PMC13382688; doi:10.1021/acsomega.6c03466)
Supplement: Supplementary file 2 [file ao6c03466_si_002.docx]

# S.2 Results and discussion

This supplementary material presents the results of competitive organic acids effect and resin regeneration experiments.

## S2.1 Adsorption tests

### S2.1.1 Effect of Competitive Organic Acids

In real fuel ethanol, there are different organic acids such as acetic, formic, butyric, and propionic acids ^9^, that may compete with each other for the active sites of the resin. The present study corroborated this finding, with similar organic acids being identified in the analyzed samples. Because of the nature of the adsorbent, they can potentially interfere with acetic acid adsorption. Therefore, a competitive adsorption experiment was performed, and the results are illustrated in Figure S1. The results indicated that acetic acid removal was more efficient when it was evaluated alone, achieving 62% adsorption. In the presence of formic acid, this rate had a slight reduction to 61%, while in the presence of propionic acid, the removal of acetic acid was 60%. The most expressive impact was observed with butyric acid, whose presence reduced acetic acid removal to 47%, suggesting a more significant competitive effect, possibly related to the higher hydrophobicity or higher molecular volume of this compound. When the three acids were added simultaneously (mixture), the removal rate was 59%, which indicates moderate interference in acetic acid adsorption.

In general, the data demonstrated that, even in the presence of other organic acids, the resin maintained good performance in acetic acid removal, with relatively discrete losses in efficiency. The greatest interference was attributed to butyric acid, corroborating previous findings of competitive adsorption between these acids on ion-exchange resins ^29^, while formic and propionic acids presented less relevant effects. These results reinforce the potential of the resin for selective applications, even in complex systems such as fuel ethanol contaminated with multiple acids.

**Figure S1.** Effect of co-existing organic acids on the acetic acid adsorption efficiency on IRA-67 resin.

### S2.1.2. Regeneration

The adsorption efficiency of acetic acid by the regenerated material was monitored over five consecutive cycles, as illustrated in Figure S2. The results show that the adsorbent maintained good performance in the first two cycles, reaching 99.3% efficiency in the second relative to the initial value (100%). From the third cycle onwards, however, the removal capacity began to decline more noticeably, with values of 86.82%, 85.74%, and 84.98% in cycles three, four, and five, respectively.

This gradual decline in efficiency may be related to partial saturation of active sites, pore obstruction, or even structural changes that occurred during repeated regeneration and reuse processes ^50^. Despite this, even after five reuses, the material still retains about 85% of its original capacity, which demonstrates good stability and quite promising performance for continuous applications.

In general, the data reinforce that regeneration using 3% NaOH solution was effective in restoring, at least partially, the functionality of the adsorbent. This points to a viable alternative in practical contexts, where material reuse is desirable without significant losses in efficiency.

**Figure S2.** Removal capacity and efficiency in the reusability of the IRA-67 resin considering five cycles of acetic acid removal.
